# Correction: Reconstructible Phylogenetic Networks: Do Not Distinguish the Indistinguishable

**DOI:** 10.1371/journal.pcbi.1007137

**Published:** 2019-06-14

**Authors:** The PLOS Computational Biology Staff

## Notice of republication

This article was republished on September 18, 2015, to correct errors that were introduced during the typesetting process. The publisher apologizes for the errors. Please download this article again to view the correct version.

## Supporting information

S1 File Republished, corrected article. (PDF)
