# Supplementary material for: Reconstructible Phylogenetic Networks: Do Not Distinguish the Indistinguishable
Source: PLoS Comput Biol. 2015 Apr 7;11(4):e1004135. doi: 10.1371/journal.pcbi.1004135 (PMC4388854; doi:10.1371/journal.pcbi.1004135)
Supplement: S1 Text — This document provides an introduction to the mathematical theory of explicit phylogenetic networks with edge lengths, leading in particular to the proofs of Propositions 1 and 2, which are necessary for the proof of Theorem 1, part (ii). In the last section, we consider networks with inheritance probabilities and their relevance for likelihood-based reconstruction. (PDF) [file pcbi.1004135.s001.pdf]

A mathematical theory of explicit phylogenetic networks with edge lengths.

This document is structured in six sections, in which we develop a theory of phylogenetic networks with edge lengths. The first section introduces the notion of isomorphism between such networks and states some obvious propositions; the second section looks in more detail at the process whereby a network displays a tree; the third shows characterizations for both the NELP and the funnel-free property; the fourth and fifth sections derive the proofs of the two propositions that are necessary for the proof of Theorem 1, part (ii). The last section shows an example relevant to likelihood frameworks modelling inheritance probabilities, in addition to edge lengths. The notation and definitions introduced in the Results section and in the Methods section within the paper will be used here. We recall in particular that, throughout this paper, networks are rooted DAGs whose leaves are bijectively labeled by taxa and whose edges have a finite set of strictly positive lengths.

Isomorphisms and sub-networks

Definition 4. Given a network $N = (V, E, \varphi, \Lambda)$, a *sub-network* $N' = (V', E', \varphi', \Lambda')$ of N is any 4-tuple such that: (a) N' is a network, (b) $V' \subseteq V$, (c) $E' \subseteq V'^2 \cap E$, (d) φ' is the restriction of φ to the taxa associated to the leaves of (V', E') and, (e) for every edge $e \in E'$, $\Lambda'(e) \subseteq \Lambda(e)$. The *union* $N_1 \cup N_2$ of two sub-networks of N, $N_1 = (V_1, E_1, \varphi_1, \Lambda_1)$, $N_2 = (V_2, E_2, \varphi_2, \Lambda_2)$, having the same root, is the sub-network $N' = (V_1 \cup V_2, E_1 \cup E_2, \varphi', \Lambda')$, where φ' is the restriction of φ to the taxa associated to the leaves of $(V_1 \cup V_2, E_1 \cup E_2)$ and for every edge $e \in E_1 \cup E_2$, $\Lambda'(e) = \Lambda_1(e) \cup \Lambda_2(e)$ (where we take the liberty to let $\Lambda_i(e) = \emptyset$ whenever $e \notin E_i$).

Note that we define the union for sub-networks of N sharing the same root, because this ensures that such union is still a network. Although these requirements could be relaxed, the definition above is sufficient for the purposes of the current paper.

Definition 5. Let $N_1 = (V_1, E_1, \varphi_1, \Lambda_1)$ and $N_2 = (V_2, E_2, \varphi_2, \Lambda_2)$ be two networks on $\mathcal{X}$. N_1 and N_2 are *isomorphic* if there exists a bijection $f : V_1 \to V_2$ (called an *isomorphism*) such that:

(i) for every $x \in \mathcal{X}$, $f(\varphi_1(x)) = \varphi_2(x)$;

(ii) for every pair of nodes $(u, v) \in V_1^2$, $(u, v) \in E_1 \Leftrightarrow (f(u), f(v)) \in E_2$ and whenever both these edges exist, $\Lambda_1((u, v)) = \Lambda_2((f(u), f(v)))$.

The following four lemmas are trivially true, and for brevity we do not include their proofs here.

Lemma 2. *Let N_1 and N_2 be isomorphic networks. Then every sub-network of N_1 is isomorphic to some sub-network of N_2.*

Definition 6. Let P be a root-leaf path of a network N. The *depth* of a node v in P is the length of the weighted path in P from the root of P to v. We say that P is *to* x, if P is a network on the set $\{x\}$, that is, if its only leaf is labelled by taxon x.

Lemma 3. *A root-leaf path P_1 is isomorphic to P_2 if and only if (a) P_2 is a root-leaf path to the same taxon as P_1, and (b) for every node v_i in any of the two root-leaf paths, say P_i, there exist a node v_j in the other root-leaf path, P_j, such that the depth of v_i in P_i is equal to the depth of v_j in P_j.*

Lemma 4. *A wishbone $W = P_1 \cup P_2$ is isomorphic to W' if and only if W' is a wishbone and can be written as $W' = P_1' \cup P_2'$, so that (a) the longest common prefix of P_1 and P_2 has the same length as the longest common prefix of P_1' and P_2', and (b) P_1' and P_2' are isomorphic to P_1 and P_2, respectively.*

Lemma 5. *A crack $K = P_1 \cup P_2$ is isomorphic to K' if and only if K' is a crack and can be written as $K' = P_1' \cup P_2'$, so that (a) the longest common prefix of P_1 and P_2 has the same length as the longest common prefix of P_1' and P_2', (b) the longest common suffix of P_1 and P_2 has the same length as the longest common suffix of P_1' and P_2', and (c) P_1' and P_2' are isomorphic to P_1 and P_2, respectively.*

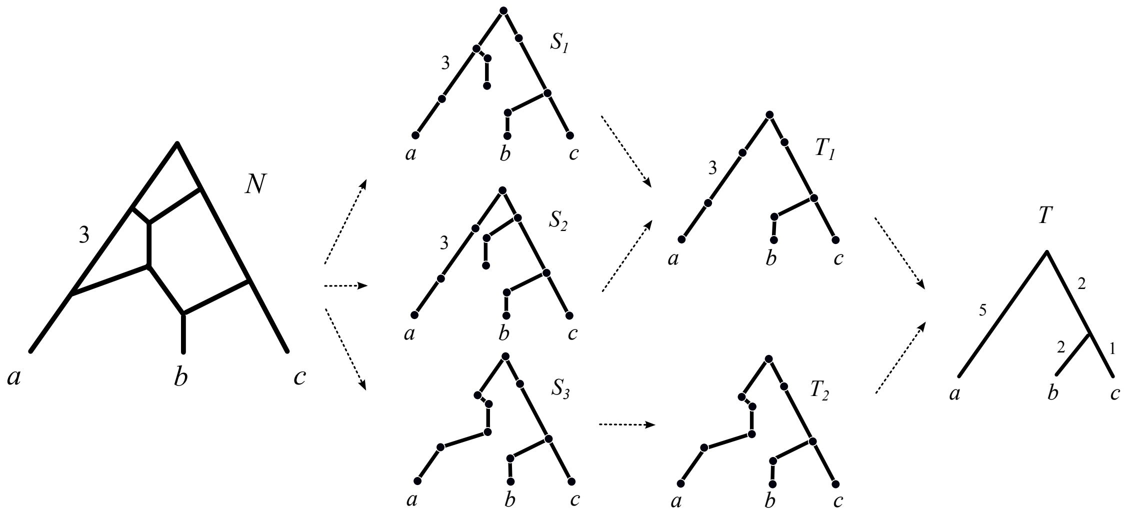

Figure S1. Illustration of the definitions of *switching* of a network, *tree contained* in a network, *tree displayed* by a network and *embeddings.* N is a network, S_1, S_2 and S_3 are three of its switchings, T_1 and T_2 are two trees contained in N, T is a tree displayed by N, and T_1 and T_2 are two embeddings of T in N. Nodes are explicitly shown in S_1, S_2, S_3, T_1 and T_2 for clarity. Unless otherwise shown, all edges are assumed to have length 1.

Switchings, trees weakly displayed and embeddings

Definition 7. Let $N = (V, E, \varphi, \Lambda)$ be a network. A *switching* $S = (V, E', \varphi, \lambda)$ of N is obtained from N by doing the following:

(i) for each node $v \in V$, delete all incoming edges of v except one; let E' be the resulting set of edges;
(ii) for each edge $e \in E'$, assign to e a single length $\lambda(e) \in \Lambda(e)$.

Note that technically a switching is not a network, as action (i) above may create leaves in S that are not labelled by any taxon. Biologically, a switching corresponds to the evolutionary tree describing the history of a single (indivisible and thus non-recombining) character carried by the root of N; some lineages in this tree may never reach any leaf of N. Moreover, since each edge in this tree corresponds to a unique path in the underlying real evolutionary history, it is clear why a switching only allows one length per edge (see Fig. 8 in the main text). Note that the trees contained by a network (defined in the Results section within the paper) can also be seen as the trees that can be obtained from a switching by removing all nodes and edges that have no descendant labelled by a taxon.

Definition 8. Let N be a network. A tree *weakly contained* in N is a tree T that is a sub-network of N and has the same root as N. A tree *weakly displayed* by N is any tree T can be obtained (up to isomorphism) by suppressing all suppressible nodes from a tree T' weakly contained in N. Tree T' is called an *embedding* of T in N. The set of trees weakly displayed by N is denoted by $\widetilde{\mathcal{T}}(N)$.

The difference with the definition of trees displayed by a network is that a network on $\mathcal{X}$ can only display trees on $\mathcal{X}$, whereas it can weakly display any tree on $\mathcal{X}'$, with $\emptyset \subset \mathcal{X}' \subseteq \mathcal{X}$. Note that if a tree T is displayed by a network N, then T is also weakly displayed by N. The definitions of switching of a network, tree contained in a network, tree displayed by a network and embeddings are illustrated in Fig. S1. We note that every switching of a network N gives rise to a unique tree contained in N and

every tree contained in N gives rise to a unique tree displayed by N. However, the converses of these two propositions are not true: several switchings of N can give rise the same tree contained in N, and several trees contained in N can give rise to the same tree displayed by N. The latter means that $T \in \mathcal{T}(N)$ can have several embeddings in N.

Lemma 6. *Let T be a tree on $\mathcal{X} = \{x_1, x_2, \ldots, x_n\}$ with no suppressible nodes. Let $P_1, P_2, \ldots, P_n$ be the root-leaf paths in T to $x_1, x_2, \ldots, x_n$, respectively. For all $i, j \in \{1, 2, \ldots, n\}$, let λ_i be the length of P_i and let λ_{ij} be the length of the longest common prefix of P_i and P_j. Any embedding of T is the union of n root-leaf paths $P'_1, P'_2, \ldots, P'_n$ to $x_1, x_2, \ldots, x_n$, such that for all $i, j \in \{1, 2, \ldots, n\}$, P'_i has length λ_i, $P'_i \cup P'_j$ is a wishbone and the length of the longest common prefix of P'_i and P'_j is λ_{ij}.*

Proof. Let T_e be an embedding of T. By definition of embedding, suppressing all suppressible nodes in T_e gives rise to a tree T' isomorphic to T. Because suppressing nodes does not change the taxon set of a tree, T_e is a tree on the same taxa as T', and thus on the same taxa as T, that is $\mathcal{X} = \{x_1, x_2, \ldots, x_n\}$. Because T_e is a tree on $\mathcal{X}$, T_e equals the union of its n root-leaf paths to $x_1, x_2, \ldots, x_n$ — which we call $P'_1, P'_2, \ldots, P'_n$ respectively — and each pair of these paths have nothing in common other than a prefix, meaning that $P'_i \cup P'_j$ is a wishbone. Now let λ'_i denote the length of P'_i and λ'_{ij} denote the length of the longest common prefix of P'_i and P'_j. It remains to prove that $\lambda'_i = \lambda_i$ and $\lambda'_{ij} = \lambda_{ij}$, for all $i, j \in \{1, 2, \ldots, n\}$.

Because suppressing nodes does not change the length of a root-leaf path nor the taxon labelling its leaf, then the root-leaf path to x_i in T', which is derived from P'_i, must have length λ'_i. But because T and T' are isomorphic, then they contain the same root-leaf paths, up to isomorphism (by Lemma 2 and Lemma 3). The (unique) root-leaf paths to x_i in T and T' are thus isomorphic and have therefore the same length, that is, $\lambda_i = \lambda'_i$.

Similarly, because suppressing nodes transforms a wishbone in another wishbone, without changing the length of the longest prefix common to its two root-leaf paths, nor the taxa labelling their leaves, then the root-leaf paths to x_i and x_j in T', which are derived from P'_i and P'_j, respectively, must form a wishbone W', with a longest common prefix of length λ'_{ij}. Because T and T' are isomorphic, then they contain the same wishbones, up to isomorphism (by Lemma 2 and Lemma 4), meaning that W' and $P_i \cup P_j$ must be isomorphic. By Lemma 4, the longest common prefix of P_i and P_j must then have length $\lambda_{ij} = \lambda'_{ij}$. □

Lemma 7. *Let T, T' denote trees and N, N' denote networks.*
(a) If N and N' are isomorphic, then $\tilde{\mathcal{T}}(N) = \tilde{\mathcal{T}}(N')$.
(b) If T' is obtained by suppressing all suppressible nodes from T, then $\tilde{\mathcal{T}}(T) = \tilde{\mathcal{T}}(T')$.
(c) If T' is an embedding of T, then $\tilde{\mathcal{T}}(T) = \tilde{\mathcal{T}}(T')$.
(d) If T is weakly contained in N, then $\tilde{\mathcal{T}}(T) \subseteq \tilde{\mathcal{T}}(N)$.
(e) If T is weakly displayed by N, then $\tilde{\mathcal{T}}(T) \subseteq \tilde{\mathcal{T}}(N)$.

Proof. ***(a)*** Let T be a tree weakly contained in N. Let f be an isomorphism between N and N'. It is easy to see that the restriction of f to the nodes of T defines an isomorphism between T and a tree T' that is weakly contained in N', meaning that N and N' must weakly contain the same trees (up to isomorphism). Therefore N and N' must weakly display the same trees. ***(b)*** Let T, and thus T', be trees on $\mathcal{Y}$. Given a nonempty subset $\mathcal{X} \subseteq \mathcal{Y}$, let $T_{\mathcal{X}}$ be the (unique) tree on $\mathcal{X}$ weakly contained in T, and let $T'_{\mathcal{X}}$ be the (unique) tree on $\mathcal{X}$ weakly contained in T'. It is easy to see that $T'_{\mathcal{X}}$ can be obtained by suppressing some suppressible nodes from $T_{\mathcal{X}}$. It follows that the tree obtained by suppressing all suppressible nodes from $T'_{\mathcal{X}}$ is the same as that obtained by suppressing all suppressible nodes from $T_{\mathcal{X}}$. This means that a tree on $\mathcal{X}$ is weakly displayed by T if and only if it is weakly displayed by T'. Since this is true for any nonempty $\mathcal{X} \subseteq \mathcal{Y}$, point (b) follows. ***(c)*** By definition, if T' is an embedding of T, then there exist a tree T_i, isomorphic to T, that can be obtained by suppressing all suppressible nodes from T'. But then, by point (a), $\tilde{\mathcal{T}}(T) = \tilde{\mathcal{T}}(T_i)$ and, by point (b), $\tilde{\mathcal{T}}(T_i) = \tilde{\mathcal{T}}(T')$. By transitivity, $\tilde{\mathcal{T}}(T) = \tilde{\mathcal{T}}(T')$. ***(d)*** If T is weakly contained in N, then every tree weakly contained in T is also weakly contained in N. Therefore

every tree weakly displayed by T is also weakly displayed by N. *(e)* If T is weakly displayed by N, then by definition there exist an embedding T' of T in N. But then, by point (c), $\tilde{\mathcal{T}}(T) = \tilde{\mathcal{T}}(T')$, and, because T' is weakly contained in N, $\tilde{\mathcal{T}}(T') \subseteq \tilde{\mathcal{T}}(N)$ (by point(d)). By transitivity, $\tilde{\mathcal{T}}(T) \subseteq \tilde{\mathcal{T}}(N)$. □

Lemma 8. *For every tree T_{wc} weakly contained in a network N, there exists a tree T_c contained in N that weakly contains T_{wc}.*

Proof. Let N be a network on $\mathcal{X}$ and T_{wc} be a tree on $\mathcal{X}' \subseteq \mathcal{X}$. Number the taxa in $\mathcal{X} \setminus \mathcal{X}'$ so that we have $\mathcal{X} \setminus \mathcal{X}' = \{x_1, x_2, \ldots, x_{|\mathcal{X}|-|\mathcal{X}'|}\}$. For convenience of notation, let $T_0 = T_{wc}$. For every $i \in \{1, 2, \ldots, |\mathcal{X}| - |\mathcal{X}'|\}$, we now show how to define T_i on $\mathcal{X}' \cup \{x_1, \ldots, x_i\}$ that weakly contains T_{i-1} and is weakly contained in N. Let P_i be a root-leaf path in N composed by the edges traversed by the following walk from the leaf labelled by x_i to the root in N: from the leaf labelled by x_i always take an edge e of N in its inverse direction (from its head to its tail), with any of e's associated lengths, until you end up in a node v belonging to T_{i-1}; from then on, follow the (inverse) path from v to the root of T_{i-1} (which coincides with that of N). By construction, P_i and T_{i-1} share a common prefix and nothing else. If we now define T_i as the union of T_{i-1} and P_i, it is clear that T_i is a tree on $\mathcal{X}' \cup \{x_1, \ldots, x_i\}$ that weakly contains T_{i-1} and is weakly contained in N. Now consider the sequence of trees $T_{wc} = T_0, T_1, \ldots, T_{|\mathcal{X}|-|\mathcal{X}'|}$. Each of these trees is weakly contained in N, and (because the relation of weak containment is transitive) weakly containing all its predecessors. Because $T_{|\mathcal{X}|-|\mathcal{X}'|}$ is a tree on $\mathcal{X}$ weakly contained in N, $T_{|\mathcal{X}|-|\mathcal{X}'|}$ is contained in N and weakly contains T_{wc}, thus concluding the proof. □

Proposition 3. *Let N and N' be networks. Then they are indistinguishable if and only if they weakly display the same trees.*

Proof. The *if* part is trivial. Suppose N and N' weakly display the same trees. Then they must be networks on the same taxon set $\mathcal{X}$, otherwise the network on the larger set of taxa, say, N, would weakly display trees with taxa that can never be present in trees weakly displayed by N'. Because N and N' weakly display the same trees on all taxon subsets $\mathcal{X}' \subseteq \mathcal{X}$, then they also display the same trees on $\mathcal{X}$, that is, they are indistinguishable.

As for the *only if* part, we prove that, assuming N and N' are indistinguishable, $\tilde{\mathcal{T}}(N) = \tilde{\mathcal{T}}(N')$. We just prove $\tilde{\mathcal{T}}(N) \subseteq \tilde{\mathcal{T}}(N')$, as the proof of $\tilde{\mathcal{T}}(N') \subseteq \tilde{\mathcal{T}}(N)$ is symmetric. Let $T \in \tilde{\mathcal{T}}(N)$ be a tree weakly displayed by N, and let T_e be an embedding of T in N. Because T_e is weakly contained in N, by Lemma 8 there exists a tree T_c contained in N that weakly contains T_e. But then, by points (c) and (d) in Lemma 7,

$$\tilde{\mathcal{T}}(T) = \tilde{\mathcal{T}}(T_e) \subseteq \tilde{\mathcal{T}}(T_c). \tag{1}$$

Now let T_d be the tree obtained by suppressing all suppressible nodes in T_c. Because T_c is contained in N, then T_d is displayed by N and thus by N', given that N and N' are indistinguishable. Therefore, by points (b) and (e) in Lemma 7,

$$\tilde{\mathcal{T}}(T_c) = \tilde{\mathcal{T}}(T_d) \subseteq \tilde{\mathcal{T}}(N'). \tag{2}$$

By putting together relations (1) and (2), we have that $T \in \tilde{\mathcal{T}}(T) \subseteq \tilde{\mathcal{T}}(N')$. Because this holds for any $T \in \tilde{\mathcal{T}}(N)$, we conclude $\tilde{\mathcal{T}}(N) \subseteq \tilde{\mathcal{T}}(N')$. □

Corollary 3. *Let N and N' be indistinguishable networks. Then, for every root-leaf path P in N, there exists in N' an equally long root-leaf path to the same taxon as P.*

Proof. Suppose there exists in N a root-leaf path to x of length λ. By suppressing all suppressible nodes from this path, one obtains a tree T consisting of one edge of length λ, whose head is labelled by x. Because N and N' are indistinguishable, they both weakly display T (by Proposition 3). But since any embedding of T must consist of a single root-leaf path to x of length λ (by Lemma 6), such a root-leaf path must exist in N'. □

Characterizing the funnel-free and the NELP properties

In this section we investigate what it means for a network to be canonical and to satisfy the NELP property. We start by characterizing the topological constraint (absence of funnels) that defines canonical networks.

Proposition 4. *A network N is funnel-free if and only if, for any two distinct non-root nodes u and w in N, there exist two node-disjoint directed paths from u and w to two distinct leaves in N.*

Proof. First, note that if N is not funnel-free, then the stated property does not hold: it suffices to take a funnel as u and its only direct descendant as w and then it is clear that any two paths from u and w cannot be node-disjoint.

Second, we prove that in a funnel-free network, for any two distinct nodes u and w, neither of which coincides with the root, there always exist two node-disjoint paths π_{ux} and π_{wy}, from u to x and from w to y respectively, where x and y are leaves of N. Let $d_N(v)$ denote the number of proper descendants (that is, not including v) of a node v in a network N. We prove our claim by induction on $d_N(u) + d_N(w)$.

First, suppose $d_N(u) + d_N(w) = 0$. In this case u and w are leaves and the thesis trivially holds.

Then, suppose $d_N(u) + d_N(w) = n > 0$. Assume, without loss of generality, that $d_N(w) \geq d_N(u)$. Then $d_N(w) > 0$, which means that w is an internal node. As a consequence, w must have at least two children nodes, because otherwise w would be a funnel or a root with outdegree 1 (which we have excluded). Of these children nodes, at least one must be different from u. Call this node w'. Because w' is a descendant of w, then $d_N(w') < d_N(w)$ and so $d_N(u) + d_N(w') < n$. By the inductive hypothesis, we can then assume that the thesis holds for the pair of (different, non-root) nodes (u, w'). That is, there exist in N two disjoint directed paths π_{ux} and $\pi_{w'y}$, from u to x and from w' to y respectively, such that x and y are leaves of N. Now form a new path π_{wy} by appending the edge (w, w') at the beginning of $\pi_{w'y}$. Note that w cannot be part of π_{ux} because otherwise w would be a descendant of u and that would contradict $d_N(w) \geq d_N(u)$. Because of this, and because $\pi_{w'y}$ and π_{ux} are disjoint, then also π_{wy} and π_{ux} are disjoint. The thesis then holds also in the inductive step and the theorem follows. □

We now examine the NELP property. It turns out that this property is equivalent to requiring the uniqueness of embeddings for all trees weakly displayed by the network. In order to prove this, we show the following trivial result that will be useful throughout this document.

Lemma 9. *A network N satisfies the NELP property if and only if, for every taxon x in N, all root-leaf paths in N to x have different lengths.*

Proof. For the *if* part, note that if N does not satisfy the NELP property, then there are two distinct weighted paths (π_1, λ_1), (π_2, λ_2) with the same lengths and endpoints. In this case it is easy to extend these paths to two distinct root-leaf paths P_1 and P_2 leading to the same taxon x and having the same length. As for the *only if* part, the existence of two root-leaf paths in N of equal lengths to the same taxon x clearly implies that N violates the NELP property. □

Proposition 5. *A network N satisfies the NELP property if and only if every tree weakly displayed by N has a unique embedding in N.*

Proof. For the *only if* part, let T be a tree on $\{x_1, x_2, \ldots, x_n\}$, weakly displayed by N, with $\lambda_1, \lambda_2, \ldots, \lambda_n$ being the lengths of the root-leaf paths to $x_1, x_2, \ldots, x_n$ in T. Suppose T_1 and T_2 are two embeddings of T in N. Then T_1 and T_2 must each consists of the union of n root-leaf paths to $x_1, x_2, \ldots, x_n$ of lengths $\lambda_1, \lambda_2, \ldots, \lambda_n$, respectively (Lemma 6). But because N satisfies the NELP property, then for any $i \in \{1, 2, \ldots, n\}$ there can be only one path to x_i of length λ_i (Lemma 9), meaning that $T_1 = T_2$.

As for the *if* part, suppose every tree displayed by a network N has a unique embedding in N, but that N does not satisfy the NELP property. Then (by Lemma 9) there exist two distinct root-leaf paths P_1 and P_2 to the same taxon (say, x) that have the same length λ. But then P_1 and P_2 are distinct

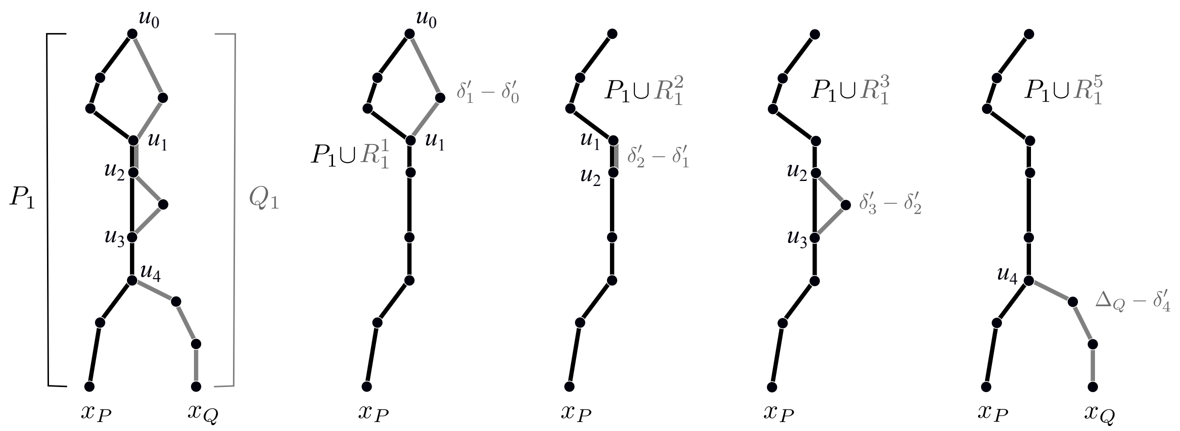

Figure S2. Two root-leaf paths P_1 and Q_1 intersecting in 5 nodes, their corresponding cracks $P_1 \cup R_1^1, P_1 \cup R_1^2, P_1 \cup R_1^3$ and wishbone $P_1 \cup R_1^5$ (see the proof of Lemma 10).

embeddings in N of the tree consisting of a single edge whose head is labelled by x, thus contradicting the uniqueness of embeddings. □

Note that the network in Fig. S1, which has several embeddings for T (namely T_1 and T_2), violates the NELP property: for example there are three different paths of length 5 from the root to the leaf labelled by a. Also note that the uniqueness of embeddings for the trees in $\mathcal{T}(N)$ does not imply the NELP property: consider for example the top-left network in Fig. 10 in the main text, where the two trees displayed by this network have unique embeddings, but the network does not satisfy the NELP property.

Proving Proposition 1

Lemma 10. *Let P_1, Q_1 be two root leaf paths in a network N_1 and let P_2 and Q_2 be two root-leaf paths respectively isomorphic to P_1 and Q_1, in another network N_2. Suppose that N_2 satisfies the NELP property and that, for every wishbone or crack K_1 contained in $P_1 \cup Q_1$ and containing P_1, there exists in N_2 a wishbone or crack K_2 isomorphic to K_1. Then P_2 and Q_2 intersect each other at the same depths as P_1 and Q_1, that is:*

P_1 and Q_1 have a node u in common that is at depth δ in P_1 and depth δ' in Q_1 if and only if P_2 and Q_2 have a node v in common that is at depth δ in P_2 and depth δ' in Q_2.

Proof. In the following, we prove that if P_1 and Q_1 have in common the nodes $u_0, u_1, \ldots, u_k$ and only these nodes (where u_0 is the root of N_1), with respective depths $\delta_0 = 0 < \delta_1 < \ldots < \delta_k$ in P_1 and depths $\delta'_0 = 0 < \delta'_1 < \ldots < \delta'_k$ in Q_1, then in N_2 there must be exactly $k+1$ nodes in common between P_2 and Q_2, and these nodes must be at depths $\delta_0, \delta_1, \ldots, \delta_k$ in P_2 and depths $\delta'_0, \delta'_1, \ldots, \delta'_k$ in Q_2. This statement is clearly equivalent to the statement that we wish to prove.

First, let $v_0, v_1, \ldots, v_k$ be the nodes that have respective depths $\delta_0, \delta_1, \ldots, \delta_k$ in P_2. The existence of these nodes is guaranteed by the fact that P_2 is isomorphic to P_1, and P_1 has nodes $u_0, u_1, \ldots, u_k$ at exactly those depths (Lemma 3). Furthermore let Δ_P be the length of P_1 and P_2, let Δ_Q be the length of Q_1 and Q_2, let x_P be the taxon labelling the leaves of P_1 and P_2 and let x_Q be the taxon labelling the leaves of Q_1 and Q_2. Below, we prove the following two claims:

(C1) For every $i \in \{1, 2, \ldots, k\}$, there exists in N_2 a weighted path from v_{i-1} to v_i of length $\delta'_i - \delta'_{i-1}$ that has no node in common with P_2 other than v_{i-1} and v_i.

(C2) If v_k is a leaf, then it is labelled by x_Q; otherwise there exists in N_2 a weighted path from v_k to the leaf labelled by x_Q that has no node in common with P_2 other than v_k and whose length is equal to $\Delta_Q - \delta'_k$.

We start with the proof of C1. See Fig. S2 in order to follow the reasoning below. First consider the trivial case where (u_{i-1}, u_i) is an edge in both P_1 and Q_1, and that it is assigned the same length λ in both of them. In this case, it is clear that $\delta_i - \delta_{i-1} = \delta'_i - \delta'_{i-1} = \lambda$. Because P_2 is isomorphic to P_1 and because u_{i-1} and u_i are consecutive nodes in P_1, the nodes at the same depths as u_{i-1} and u_i in P_2, that is v_{i-1} and v_i, must also be consecutive in P_2 (by Lemma 3). That is, (v_{i-1}, v_i) is an edge of P_2. Its length in P_2 must be equal to the difference between the depths of v_{i-1} and v_i in P_2, that is $\delta_i - \delta_{i-1} = \delta'_i - \delta'_{i-1}$. Edge (v_{i-1}, v_i) along with its length in P_2 constitutes a weighted path from v_{i-1} to v_i of length $\delta'_i - \delta'_{i-1}$ that has no node in common with P_2 other than v_{i-1} and v_i, thus proving C1 in this trivial case. In all other cases, define R_1^i as the root-leaf path that shares with P_1 its prefix down to u_{i-1} and its suffix from u_i onwards, and shares with Q_1 its portion between u_{i-1} and u_i. By construction, Q_1 has no node in common with P_1 between u_{i-1} and u_i, meaning that R_1^i and P_1 have in common only a prefix and a suffix, and thus that $P_1 \cup R_1^i$ is a crack. Because $P_1 \cup R_1^i$ is a crack containing P_1 and contained in $P_1 \cup Q_1$, then there exists in N_2 a crack isomorphic to $P_1 \cup R_1^i$. Because isomorphic cracks are the union of isomorphic root-leaf paths (Lemma 5) and because in N_2 there can be no root-leaf path isomorphic to P_1 other than P_2 (as N_2 satisfies the NELP property), then the crack isomorphic to $P_1 \cup R_1^i$ in N_2 can be written as $P_2 \cup R_2^i$, where R_2^i is a root-leaf path isomorphic to R_1^i. Now note that P_1 and R_1^i have lengths Δ_P and $\Delta_P - (\delta_i - \delta_{i-1}) + (\delta'_i - \delta'_{i-1})$, respectively, and longest common prefix and suffix of lengths δ_{i-1} and $\Delta_P - \delta_i$, respectively (as δ_{i-1} and δ_i are the respective depths of u_{i-1} and u_i in P_1). By Lemma 5, also P_2 and R_2^i and their longest common prefix and suffix must have these lengths. This implies that R_2^i separates from P_2 at the node at depth δ_{i-1} in P_2 — which by construction is v_{i-1} — then follows a weighted path of length $\delta'_i - \delta'_{i-1}$ that has no node in common with P_2 other than its extremes, and finally joins up with P_2 at the node at depth δ_i in P_2 — which by construction is v_i. The portion of R_2^i between v_{i-1} and v_i has length $\delta'_i - \delta'_{i-1}$ and no node in common with P_2 other than v_{i-1} and v_i, thus proving claim C1.

As for C2, if v_k is a leaf, since v_k belongs to both P_2 and Q_2, then it is the leaf of both P_2 and Q_2, and thus it must be labelled by $x_P = x_Q$. If instead v_k is not a leaf, then it must have strict descendants in P_2. Then, because P_1 and P_2 are isomorphic, also u_k (the node at the same depth in P_1 as v_k in P_2) must have strict descendants and is thus not a leaf. Define then R_1^{k+1} as the root-leaf path that shares with P_1 its prefix down to u_k, and shares with Q_1 its suffix from u_k to the leaf labelled by x_Q (as u_k is not a leaf, this suffix contains at least one edge). By construction, Q_1 has no node in common with P_1 after separating from it in u_k, meaning that R_1^{k+1} and P_1 have in common only a prefix, and thus that $P_1 \cup R_1^{k+1}$ is a wishbone. Because $P_1 \cup R_1^{k+1}$ is a wishbone containing P_1 and contained in $P_1 \cup Q_1$, then there exists in N_2 a wishbone isomorphic to $P_1 \cup R_1^{k+1}$. Because isomorphic wishbones are the union of isomorphic root-leaf paths (Lemma 4) and because in N_2 there can be no root-leaf path isomorphic to P_1 other than P_2 (as N_2 satisfies the NELP property), then the crack isomorphic to $P_1 \cup R_1^{k+1}$ in N_2 can be written as $P_2 \cup R_2^{k+1}$, where R_2^{k+1} is a root-leaf path isomorphic to R_1^{k+1}. Now note that P_1 and R_1^{k+1} have lengths Δ_P and $\Delta_Q - \delta'_k + \delta_k$, respectively, and longest common prefix of length δ_k. By Lemma 4, also P_2 and R_2^{k+1} and their longest common prefix must have these lengths. This implies that R_2^{k+1} separates from P_2 at the node at depth δ_k in P_2 — which by construction is v_k — and then follows a weighted path of length $\Delta_Q - \delta'_k$, that has no node in common with P_2 other than v_k, and that ends up in a leaf labelled by x_Q (as R_2^{k+1} is isomorphic to R_1^{k+1}). Thus C2 is also proved.

As a consequence of C1 and C2, one can construct a root-leaf path R in N_2 by concatenating all the weighted paths whose existence has been proven in C1 and C2. Clearly, R is a root-leaf path to x_Q and it has a total length of Δ_Q, as:

$$\begin{cases} \sum_{i=1}^{k}(\delta'_i - \delta'_{i-1}) = \delta'_k = \Delta_Q & \text{if } v_k \text{ is a leaf,} \\ \Delta_Q - \delta'_k + \sum_{i=1}^{k}(\delta'_i - \delta'_{i-1}) = \Delta_Q & \text{otherwise.} \end{cases}$$

Because in N_2 there can be no root-leaf path to x_Q of length Δ_Q other than Q_2 (as N_2 satisfies the NELP property), then $R = Q_2$. Now note that C1 and C2 imply that the only nodes that $R = Q_2$ has in common with P_2 are $v_0, v_1, \ldots, v_k$. Moreover, for every $i \in \{0, 1, \ldots, k\}$, the depth of v_i in P_2 equals δ_i (by definition), and the depth of v_i in $R = Q_2$ equals $\sum_{j=1}^{i}(\delta'_j - \delta'_{j-1}) = \delta'_i$, which is what we set out to prove. □

Proposition 1. *Two networks N_1 and N_2 with the NELP property are isomorphic if and only if they contain the same wishbones and cracks (up to isomorphism).*

Proof. The *only if* part is trivial: if N_1 and N_2 are isomorphic, than each wishbone or crack W contained in one of the two networks must have an isomorphic sub-network W' in the other (by Lemma 2), and W' must be either be a wishbone or a crack (by Lemmas 4 and 5).

As for the *if* part, let us now suppose networks $N_1 = (V_1, E_1, \varphi_1, \Lambda_1)$ and $N_2 = (V_2, E_2, \varphi_2, \Lambda_2)$ satisfy the NELP property and contain isomorphic wishbones and cracks. We prove that N_1 and N_2 are isomorphic.

Because N_1 and N_2 contain the same wishbones and cracks (up to isomorphism), and because a root-leaf path (which is both a wishbone and a crack) can only be isomorphic to another root-leaf path (Lemma 3), then N_1 and N_2 must contain the same root-leaf paths (up to isomorphism). Note that N_1 and N_2 must be networks on the same taxon set $\mathcal{X}$, because otherwise any root-leaf path that leads to a leaf corresponding to a taxon present in only one of the networks would have no isomorphic root-leaf path in the other network. Then, define a relation $\sim$ between V_1 and V_2 as follows: for any $v_1 \in V_1$ and $v_2 \in V_2$ we write $v_1 \sim v_2$ if there exist two isomorphic root-leaf paths P_1 and P_2, in N_1 and N_2, respectively, that contain v_1 and v_2, respectively, such that v_1 has the same depth in P_1 as v_2 in P_2. Note that, because N_1 and N_2 contain isomorphic root-leaf paths, for every $v_1 \in V_1$ there exists a $v_2 \in V_2$ such that $v_1 \sim v_2$ (by Lemma 3). Moreover, we now prove that such v_2 is unique. Suppose there exist v_2 and v'_2 such that $v_1 \sim v_2$ and $v_1 \sim v'_2$. This would mean (see Fig. S3) that there exist two isomorphic root-leaf paths P_1 and P_2, in N_1 and N_2, respectively, that have v_1 and v_2 at the same depth δ and that there exist two isomorphic root-leaf paths Q_1 and Q_2, in N_1 and N_2, respectively, that have v_1 and v'_2 at the same depth δ'. Because N_1 and N_2 contain the same wishbones and cracks, in particular for every wishbone or crack contained in $P_1 \cup Q_1$ and containing P_1 there exists an isomorphic wishbone or crack in N_2. Thus the assumptions of Lemma 10 are verified, meaning that P_2 and Q_2 must intersect each other at the same depths as P_1 and Q_1. As a result, because P_1 and Q_1 have a node v_1 in common that is at depth δ in P_1 and depth δ' in Q_1, then P_2 and Q_2 must have a node in common that is at depth δ in P_2 and depth δ' in Q_2. But v_2 and v'_2 are precisely the nodes in P_2 and Q_2 at depths δ and δ', respectively, meaning that we must have $v_2 = v'_2$. We thus conclude that there is a unique $v_2 \in V_2$ such that $v_1 \sim v_2$. Similarly, for every $v_2 \in V_2$ there exists a unique $v_1 \in V_1$ such that $v_1 \sim v_2$. Thus, relation $\sim$ identifies a bijection $f : V_1 \to V_2$, defined by $f(v_1) = v_2 \Leftrightarrow v_1 \sim v_2$.

We now prove that bijection f is an isomorphism between N_1 and N_2, by showing that it verifies the two requirements in Definition 5. First, note that for every $x \in \mathcal{X}$ we can consider a root-leaf path P_1 in N_1 ending in $\varphi_1(x)$ and its isomorphic equivalent P_2 in N_2 ending in $\varphi_2(x)$. Because the two paths are isomorphic, $\varphi_1(x)$ must lie at the same depth in P_1 as $\varphi_2(x)$ in P_2. Thus $\varphi_1(x) \sim \varphi_2(x)$, that is $f(\varphi_1(x)) = \varphi_2(x)$.

Second, we show that if $e_1 = (u, v) \in E_1$ is an edge of N_1 having a length $\lambda \in \Lambda_1(e_1)$ then $(f(u), f(v))$ is an edge of N_2 having also length λ. Let P_1 be any root-leaf path that passes via e_1 and assigns length λ to e_1. Let δ and $\delta + \lambda$ be the depths of u and v, respectively, in P_1. Because N_1 and N_2 contain isomorphic root-leaf paths, N_2 must contain a root-leaf path P_2, isomorphic to P_1. As a consequence of Lemma 3, P_2 must have two nodes at depths δ and $\delta + \lambda$ — which by construction must be $f(u)$ and $f(v)$, respectively — connected by an edge $e_2 = (f(u), f(v)) \in E_2$ having length λ in P_2, that is, $\lambda \in \Lambda_2(e_2)$. This allows to conclude that $\Lambda_1((u, v)) \subseteq \Lambda_2((f(u), f(v)))$. Similarly, we can prove that $\Lambda_2((f(u), f(v))) \subseteq \Lambda_1((u, v))$, which allows us to conclude that f satisfies point (ii) in Definition 5. Bijection f is thus an isomorphism

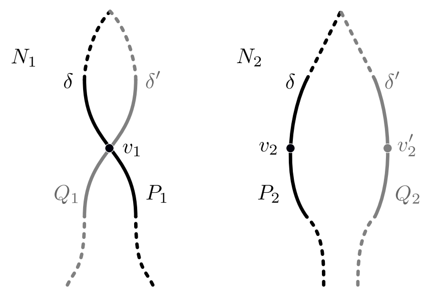

Figure S3. Illustration for the proof of Proposition 1.

between N_1 and N_2. □

Proving Proposition 2

In order to prove that two indistinguishable funnel-free networks with the NELP property have isomorphic wishbones and cracks, we need some more accessory results and notation.

Definition 9. Let P and Q be two root-leaf paths in a network N. We denote the length of the longest common prefix of P and Q by $\mu(P,Q)$. We say that P and Q *separate* at node v, if $P \neq Q$, and v is the last node in the longest common prefix of P and Q. Finally, P and Q *separate openly* at v if they separate at v and the direct descendants of v in P and Q are distinct nodes.

Note that if the longest common prefix of P and Q consists of only one node, then $\mu(P,Q) = 0$. In general, if P and Q separate at v, then the depth of v in P and Q is precisely $\mu(P,Q)$. Moreover, note that two distinct root-leaf paths P and Q do not separate openly at v when they contain the same edge (v,v'), but with different lengths in P and Q.

Definition 10. Let P and Q denote root-leaf paths. An *open crack* is a crack $P \cup Q$ where P and Q separate openly. A *closed crack* is a crack $P \cup Q$ where P and Q differ for the length of exactly one edge.

Note that all cracks are either open, closed or root-leaf paths. In Fig. 14 in the main text, S and R are an open crack and a closed crack, respectively.

Lemma 11. *(Three-prefix condition). Let P_1, P_2, P_3 be root-leaf paths in a network N. Then the two smallest of $\mu(P_1,P_2)$, $\mu(P_1,P_3)$, $\mu(P_2,P_3)$ are equal, or, equivalently, for $\{i,j,k\} = \{1,2,3\}$:*

$$\mu(P_i,P_j) \geq \min\{\mu(P_i,P_k), \mu(P_j,P_k)\}.$$

Proof. First note that the two smallest of three numbers x_1, x_2, x_3 are equal if and only if $x_i \geq \min\{x_j, x_k\}$, for $\{i,j,k\} = \{1,2,3\}$: without loss of generality suppose $x_1 \leq x_2 \leq x_3$ and note that — while $x_2 \geq \min\{x_1,x_3\} = x_1$ and $x_3 \geq \min\{x_1,x_2\} = x_1$ are trivially true — $x_1 \geq \min\{x_2,x_3\}$ holds if and only if $x_1 = x_2$. In order to prove that the two smallest of $\mu(P_1,P_2)$, $\mu(P_1,P_3)$, $\mu(P_2,P_3)$ are equal, imagine following P_1, P_2 and P_3 from the root until at least one of them separates from the others: let v be a node at depth δ in P_1, P_2, P_3, where either only P_i separates from P_j and P_k, or all three P_1, P_2, P_3 separate. In both cases, $\delta = \mu(P_i,P_j) = \mu(P_i,P_k) \leq \mu(P_j,P_k)$, thus concluding the proof of this lemma. □

Lemma 12. *Let N and N' be two indistinguishable funnel-free networks, and let N' satisfy the NELP property. Let P_1 and P_2 be root-leaf paths in N, whose union $P_1 \cup P_2$ is a wishbone. Let P_1' and P_2'*

be the root-leaf paths in N' to the same taxa as P_1 and P_2, and having the same lengths as P_1 and P_2, respectively (whose existence and uniqueness are guaranteed by Corollary 3 and Lemma 9, respectively). Then, $P_1' \cup P_2'$ is a wishbone of N', and

$$\mu(P_1', P_2') = \mu(P_1, P_2).$$

Proof. Let x_1 and x_2 be the taxa labelling the leaves of P_1 and P_2 (and thus P_1' and P_2'), respectively, and let λ_1 and λ_2 be the lengths of P_1 and P_2 (and thus P_1' and P_2'). Note that we may have $x_1 = x_2$, and $\lambda_1 = \lambda_2$, if $P_1 = P_2$. Because N and N' are indistinguishable, they must both weakly display T_W, the tree obtained by suppressing all suppressible nodes in the wishbone $W = P_1 \cup P_2$ (by Proposition 3). T_W must be the union of two root-leaf paths to x_1 and x_2, of lengths λ_1 and λ_2, respectively, and having a longest common prefix of length $\mu(P_1, P_2)$ (because these properties are not lost by suppressing suppressible nodes). Then, the embedding of T_W in N' must be a wishbone W' consisting of the union of two root-leaf paths to x_1 and x_2, of lengths λ_1 and λ_2, respectively, whose longest common prefix has length $\mu(P_1, P_2)$ (by Lemma 6). Because P_1' and P_2' are the unique root-leaf paths in N' to x_1 and x_2, and of lengths λ_1 and λ_2, respectively, this implies $W' = P_1' \cup P_2'$ and the lemma follows. □

Note that the lemma that we just proved includes the trivial case where $P_1 = P_2$.

Lemma 13. *Let N and N' be two indistinguishable funnel-free networks, and let N' satisfy the NELP property. Let P and Q be distinct root-leaf paths in N, whose union $P \cup Q$ is a crack. Let P' and Q' be the root-leaf paths in N' to the same taxa, and having the same lengths as P and Q, respectively (whose existence and uniqueness are guaranteed by Corollary 3 and Lemma 9, respectively). Then*

$$\mu(P', Q') \begin{cases} = \mu(P, Q) & \textit{if } P \cup Q \textit{ is an open crack,} \\ \geq \mu(P, Q) & \textit{if } P \cup Q \textit{ is a closed crack.} \end{cases}$$

Proof. The proof is by induction on the number of edges in the longest suffix common to P and Q. Throughout this proof, for any root-leaf path X in N, let X' denote the root-leaf path in N' to the same taxon and having the same length as X. Moreover, let u be the last node in the longest prefix common to P and Q and let v be the first node in the longest suffix common to P and Q. Because $P \neq Q$, u must be a strict ancestor of v. Moreover, let u_P and u_Q be the direct ancestors of v in P and Q, respectively. Of these two nodes, at least one is not a strict ancestor of the other, otherwise N would contain a cycle. Thus, without loss of generality, we assume throughout that u_Q is not a strict ancestor of u_P. Note that if $P \cup Q$ is an open crack, at least one between u_P and u_Q must be different from u. Because we require that u_Q is not a strict ancestor of u_P, it follows that $u_Q \neq u$, when $P \cup Q$ is an open crack. Finally, in the particular case where $P \cup Q$ is a closed crack and $u = u_Q$ is the root of N, the statement trivially holds, as $\mu(P, Q) = 0 \leq \mu(P', Q')$. Thus, we assume throughout that u_Q is not the root of N. Because it cannot be a leaf or a funnel, either, we can assume that u_Q has outdegree 2 or more.

Base case. (See Fig. S4, left, to follow the argument below.) Suppose that the longest suffix common to P and Q contains no edge, or, equivalently, that it consists of just a leaf v. Because u_Q has outdegree 2 or more, we can define a root-leaf path R in N that separates openly from Q at u_Q: let R have a common prefix with Q consisting of the portion of Q from the root down to u_Q; then, let R take an edge (u_Q, w) with $w \neq v$ (with any of this edge's lengths in N) and finally let R take any weighted path from w that does not end up in v (which is possible because N is funnel-free). Clearly,

$$\mu(P, Q) = \mu(P, R) \leq \mu(Q, R). \tag{3}$$

Note that R and Q have no node in common below u_Q — as otherwise R would contain v, which we excluded by construction — meaning that $Q \cup R$ is a wishbone. Moreover, $P \cup R$ is also a wishbone: assuming otherwise would mean that R has a node in common with P below u, either contradicting the

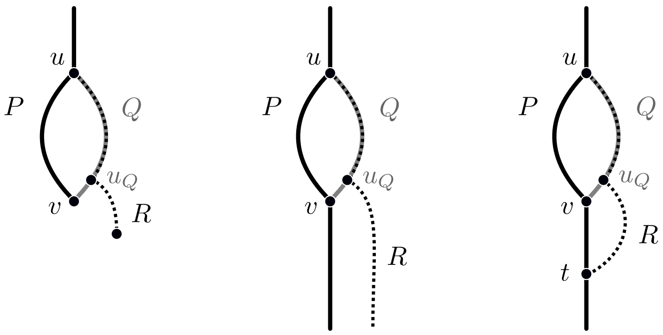

Figure S4. Illustration of the base case (left) and the inductive step (other two drawings) in the proof of Lemma 13. P is shown in black, the portion of Q not coinciding with P is shown in grey, and the portion of R not coinciding with P is represented by the dashed line. Note that, although the drawing shows the case of an open crack with $u \neq u_Q$, the proof also considers the case where $u = u_Q$, i.e. when $P \cup Q$ is a closed crack.

fact that P and Q have no nodes in common between u and v, or contradicting the requirement that u_Q is not a strict ancestor of u_P, or contradicting the requirement that v does not belong to R. Because $Q \cup R$ and $P \cup R$ are wishbones, then (by Lemma 12)

$$\mu(Q', R') = \mu(Q, R), \qquad \mu(P', R') = \mu(P, R) \tag{4}$$

Now consider two cases: either $P \cup Q$ is an open crack or it is closed. If it is open, then $u \neq u_Q$ (see above) and thus we have $\mu(P, R) < \mu(Q, R)$ in (3). Then, because of the equalities in (4), we have $\mu(P', R') < \mu(Q', R')$. But then, because of the three-prefix condition (Lemma 11), we must have $\mu(P', Q') = \mu(P', R')$. Combine this with Equations (3) and (4), to show that $\mu(P', Q') = \mu(P, Q)$. If instead $P \cup Q$ is a closed crack, then $u = u_Q$ and thus we have $\mu(P, R) = \mu(Q, R)$. Then, because of the equalities in (4), we have $\mu(P', R') = \mu(Q', R')$. In this case, the three-prefix condition (Lemma 11) implies $\mu(P', Q') \geq \mu(P', R') = \mu(Q', R')$ and thus $\mu(P', Q') \geq \mu(P, Q)$.

Inductive step. (See the two drawings on the right in Fig. S4 to follow the argument below.) Now suppose that the longest suffix common to P and Q contains $k > 0$ edges, and assume that the lemma's statements hold for every pair of root-leaf paths whose union is a crack and whose longest common suffix contains fewer than k edges. Because u_Q has outdegree 2 or more, we can define a root-leaf path R in N that separates openly from Q at u_Q: let R have a common prefix with Q consisting of the prefix of Q from the root down to u_Q; then, let R take an edge (u_Q, w) with $w \neq v$ (with any of this edge's lengths in N) and then let R continue taking edges always avoiding ending up in v (recall that N is funnel-free), until it either arrives in a leaf or in a node t belonging to $P \cup Q$. Note that such t must be a strict descendant of v in the suffix common to P and Q. (By construction $t \neq v$; moreover, if t belonged to Q and not to P, then t would lie between u_Q and v in Q, which contradicts the assumption that u_Q is a direct ancestor of v in Q; finally, if t belonged to P and not to Q, then t would either coincide with u_P or be a strict ancestor of u_P, implying that u_Q is an ancestor of u_P, which is not possible by construction.) Finally, from t onwards, let R coincide with the suffix common to P and Q. Clearly, as in the proof of the base case, the following holds:

$$\mu(P, Q) = \mu(P, R) \leq \mu(Q, R). \tag{5}$$

If R arrives in a leaf without hitting a node in $P \cup Q$ (i.e., without crossing neither P or Q), then $Q \cup R$ and $P \cup R$ are wishbones. If instead R hits $P \cup Q$ in t, then $Q \cup R$ and $P \cup R$ are open cracks: they are cracks, because, by construction, t is the first node that R has in common with P and Q after separating

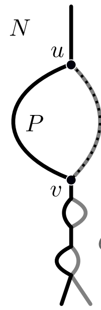

Figure S5. Illustration of the inductive case in the proof of Lemma 14. P is shown in black, the portion of Q not coinciding with P is shown in grey, and the portion of R not coinciding with P is represented by the dashed line.

from them (at u and u_Q, respectively), and because R has its suffix following t in common with P and Q; they are open because the edge (u_Q, w) in R does not belong to neither Q or P. Moreover, these open cracks are such that the two root-leaf paths that compose them have a longest common suffix of fewer than k edges, as t is a strict descendant of v. Irrespective of $Q \cup R$ and $P \cup R$ being wishbones or open cracks, we then have (by Lemma 12 or by inductive hypothesis) that

$$\mu(Q', R') = \mu(Q, R), \qquad \mu(P', R') = \mu(P, R). \tag{6}$$

Now consider two cases: either $P \cup Q$ is an open crack or it is closed. If it is open, then $u \neq u_Q$ (see above) and thus we have $\mu(P, R) < \mu(Q, R)$ in (5). Then, because of the equalities in (6), we have $\mu(P', R') < \mu(Q', R')$. But then, because of the three-prefix condition (Lemma 11), we must have $\mu(P', Q') = \mu(P', R')$. If we combine this with Equations (5) and (6), then we deduce that $\mu(P', Q') = \mu(P, Q)$. If instead $P \cup Q$ is a closed crack, then $u = u_Q$ and thus we have $\mu(P, R) = \mu(Q, R)$. Then, because of the equalities in (6), we have $\mu(P', R') = \mu(Q', R')$. In this case, the three-prefix condition (Lemma 11) implies $\mu(P', Q') \geq \mu(P', R') = \mu(Q', R')$ and thus $\mu(P', Q') \geq \mu(P, Q)$. □

Lemma 14. *Let N and N' be two indistinguishable funnel-free networks, and let N' satisfy the NELP property. Let P and Q be any root-leaf paths in N, and let P' and Q' be the root-leaf paths in N' to the same taxa, and having the same lengths as P and Q, respectively (whose existence and uniqueness are guaranteed by Corollary 3 and Lemma 9, respectively). Then, (i)*

$$\mu(P', Q') \begin{cases} = \mu(P, Q) & \text{if } P = Q \text{ or if they separate openly,} \\ \geq \mu(P, Q) & \text{otherwise.} \end{cases}$$

Moreover, (ii) if P and Q separate openly, then P' and Q' also separate openly.

Proof. Once again, throughout this proof, for any root-leaf path X in N, we let X' denote the root-leaf path in N' to the same taxon and having the same length as X. Let $\Delta(P, Q)$ denote the number of edges in $P \cup Q$ that are either present in only one of P and Q, or that are present in both, but with different lengths. We prove part (i) by induction on $\Delta(P, Q)$.

Base case. If $\Delta(P, Q) = 0$, then $P = Q$ and $P' = Q'$, trivially implying that both $\mu(P, Q)$ and $\mu(P', Q')$ equal the length of these paths, and thus $\mu(P', Q') = \mu(P, Q)$.

Inductive step. If $\Delta(P, Q) > 0$, then P and Q must separate at a node u. If they separate openly and do not have any node in common after u, then they form a wishbone, meaning that $\mu(P', Q') = \mu(P, Q)$ (by Lemma 12), thus verifying the statement. In all other cases, let v be the first node that P and Q have

in common after u. Define a new root-leaf path R that coincides with P and Q along all their common prefix down to u, then coincides with Q along the weighted path in Q from u to v, and finally coincides with P along all its suffix from v to the leaf in P (see Fig. S5). Clearly,

$$\mu(P,Q) = \mu(P,R) < \mu(Q,R). \tag{7}$$

Note that $\Delta(Q,R) = \Delta(P,Q) - \Delta(P,R)$. Because $P \neq R$, then $\Delta(P,R) > 0$. Therefore, we have $\Delta(Q,R) < \Delta(P,Q)$ and we can assume, by inductive hypothesis:

$$\mu(Q',R') \geq \mu(Q,R). \tag{8}$$

Because P and R are node-disjoint between u and v, and coincide everywhere else, clearly they form a crack. This crack is open or closed, depending on whether P and Q separate openly or by taking the same edge (u,v) with different lengths. Consider these two cases separately.

If P and Q separate openly, then $P \cup R$ is an open crack and Lemma 13 implies that

$$\mu(P',R') = \mu(P,R). \tag{9}$$

Now combine Equations (7), (8), (9) to show that $\mu(P',R') < \mu(Q',R')$. But then, because of the three-prefix condition (Lemma 11), we must have $\mu(P',Q') = \mu(P',R')$, which together with Equations (7) and (9) implies $\mu(P',Q') = \mu(P,Q)$, when P and Q separate openly.

If instead P and Q separate by taking the same edge (u,v) with different lengths, then $P \cup R$ is a closed crack. Then, by Lemma 13,

$$\mu(P',R') \geq \mu(P,R). \tag{10}$$

Now combine Equations (7), (8), (10) to show that $\mu(P,Q) \leq \mu(Q',R')$ and $\mu(P,Q) \leq \mu(P',R')$. But then, because of the three-prefix condition (Lemma 11), we must have

$$\mu(P',Q') \geq \min\{\mu(Q',R'), \mu(P',R')\} \geq \mu(P,Q),$$

which concludes our proof by induction of part (i).

As for part (ii), suppose that P and Q separate openly at a node u, at depth α in their common prefix, by taking two distinct edges (u, w_P) and (u, w_Q). Because N is funnel-free, because w_P and w_Q are distinct, and because neither of them is the root of N, then there exist two node-disjoint directed paths π_P and π_Q from w_P and w_Q, respectively, to two leaves of N (by Proposition 4). Let R_P be any root-leaf path that coincides with P along its prefix down to w_P and then follows π_P by taking its edges with any of their lengths in N. Similarly, let R_Q be any root-leaf path that coincides with Q along its prefix down to w_Q and then follows π_Q by taking its edges with any of their lengths in N. Because R_P and R_Q coincide with P and Q down to node u, and all their nodes that are strict descendants of u belong to (the node-disjoint paths) π_P and π_Q, respectively, then $R_P \cup R_Q$ is a wishbone in N, with $\mu(R_P, R_Q) = \alpha$. By Lemma 12, also $R'_P \cup R'_Q$ is a wishbone in N' and $\mu(R'_P, R'_Q) = \alpha$.

Moreover, because, by construction, $\mu(P, R_P) > \alpha$ and $\mu(Q, R_Q) > \alpha$, and because, by part (i) of the present lemma, $\mu(P', R'_P) \geq \mu(P, R_P)$ and $\mu(Q', R'_Q) \geq \mu(Q, R_Q)$, then we have

$$\mu(P', R'_P) > \alpha \qquad \text{and} \qquad \mu(Q', R'_Q) > \alpha.$$

These two relationships, together with the fact that R'_P and R'_Q separate openly at depth α, imply that P' and Q' also separate openly at depth α: if we let u' be the node at depth α in R'_P and R'_Q, the successors of u' in R'_P and R'_Q must be distinct, and belonging to P' and Q', respectively, thus implying that P' and Q' separate openly. □

The lemmas above only require one of the two networks to verify the NELP property. In the rest of this section, we concentrate on the case where both networks satisfy the NELP property, as this is among the hypotheses of Proposition 2.

Lemma 15. *Let N and N' be two indistinguishable funnel-free networks satisfying the NELP property. Let P and Q be any root-leaf paths in N, and let P' and Q' be the root-leaf paths in N' to the same taxa, and having the same lengths as P and Q, respectively (whose existence and uniqueness are guaranteed by Corollary 3 and Lemma 9, respectively). Then, P' and Q' separate openly if and only if P and Q separate openly, and*

$$\mu(P', Q') = \mu(P, Q).$$

Proof. Apply Lemma 14 both to P and Q in N and to P' and Q' in N', showing that $\mu(P', Q') \geq \mu(P, Q)$ and $\mu(P, Q) \geq \mu(P', Q')$, respectively (and thus $\mu(P', Q') = \mu(P, Q)$) and that if one pair of root-leaf paths separates openly, also the other separates openly. □

Lemma 16. *Let N and N' be two indistinguishable funnel-free networks satisfying the NELP property. Then N and N' have the same root-leaf paths (up to isomorphism). Moreover, for each root-leaf path P in one of the two networks, the root-leaf path P' isomorphic to P in the other network is unique.*

Proof. Let P be a root-leaf path in one of the two networks, say (without loss of generality), N, and let P' be the unique root-leaf path in N' to the same taxon and having the same length as P (whose existence and uniqueness are guaranteed by Corollary 3 and Lemma 9, respectively). For any node v in P, there exists a node v' at the same depth in P': this is trivial when v is at depth 0; otherwise, if we let Q be a root-leaf path that separates openly from P at v (which exists because N is funnel-free), then Q' — the root-leaf path in N' to the same taxon and having the same length as Q — must separate from P' at a node at the same depth $\mu(P', Q') = \mu(P, Q)$ as v (by Lemma 14). Symmetrically, for any node in P' there exists a node at the same depth in P. Thus, because P and P' are root-leaf paths to the same taxon and have nodes at the same depths, then P and P' are isomorphic (by Lemma 3). In conclusion, for any root-leaf path P in one of the two networks, there exists a unique isomorphic root-leaf path P' in the other, which is what we wanted to prove. □

Lemma 17. *Let N and N' be two indistinguishable funnel-free networks satisfying the NELP property. Let P_1 and P_2 be root-leaf paths in N, whose union $W = P_1 \cup P_2$ is a wishbone. Let P_1' and P_2' be the unique root-leaf paths in N' isomorphic to P_1 and P_2, respectively (Lemma 16). Then $W' = P_1' \cup P_2'$ is a wishbone in N' isomorphic to W.*

Proof. By Lemma 12, $W' = P_1' \cup P_2'$ is a wishbone with $\mu(P_1', P_2') = \mu(P_1, P_2)$. Because W and W' are wishbones, and equal to the union of pairs of isomorphic root-leaf paths, with longest common prefixes of the same length, then W and W' are isomorphic (by Lemma 4). □

Corollary 4. *Let N and N' be two indistinguishable funnel-free networks satisfying the NELP property. Then N and N' have the same wishbones (up to isomorphism).*

Proof. Apply Lemma 17 to all wishbones in N and N'. □

Lemma 18. *Let N and N' be two indistinguishable funnel-free networks with the NELP property. Then they have the same cracks (up to isomorphism).*

Proof. We prove that for any crack $K = P_1 \cup P_2$ contained in one of the two networks, there exists a crack K', isomorphic to K, in the other network. Without loss of generality, we assume here that K is a crack in N, but all arguments hold symmetrically for the case where K is a crack in N'.

We introduce some notation that is useful throughout the proof. For any root-leaf path X in N, let X' denote the unique root-leaf path in N' that is isomorphic to X (Lemma 16). The thesis is trivial when $P_1 = P_2$: in this case K is a root-leaf path, and K' is isomorphic to it. Thus we assume throughout that $P_1 \neq P_2$. Now let u be the last node in the longest prefix common to P_1 and P_2 and let v be the first node in the longest suffix common to P_1 and P_2. Because $P_1 \neq P_2$, u must be a strict ancestor of v. Also, let $\alpha = \mu(P_1, P_2) = \mu(P_1', P_2')$ (the equality is guaranteed by Lemma 15), meaning that P_1' and P_2'

separate at a node u' that has the same depth α in P_1' and P_2' as u in P_1 and P_2. Moreover let β_1 and β_2 be the (strictly positive) lengths of the weighted paths from u to v within P_1 and P_2, respectively, and let $\gamma \geq 0$ be the length of the longest suffix common to P_1 and P_2. Because v has depth $\alpha + \beta_1$ and $\alpha + \beta_2$ in P_1 and P_2, respectively, there must exist in N' two nodes v_1' and v_2' at depths $\alpha + \beta_1$ and $\alpha + \beta_2$ in P_1' and P_2', respectively. Finally, note that because P_1 and P_2 have a common suffix, they are root-leaf paths to the same taxon. Therefore also P_1' and P_2' are root-leaf paths to the same taxon, and thus end up in the same leaf. Thus P_1' and P_2' must share at least one node after separating at u'. Let v' be the first node that P_1' and P_2' have in common after separating at u' (thus a strict descendant of u'). All these notations are shown in Fig. S6.

We now prove that $v' = v_1' = v_2'$ implies that $K' = P_1' \cup P_2'$ is a crack isomorphic to K. First, note that the suffixes of P_1' and P_2' following v_1' and v_2', respectively, are weighted paths of length γ (because P_1' and P_2' have lengths $\alpha + \beta_1 + \gamma$ and $\alpha + \beta_2 + \gamma$, respectively), ending up in the same leaf. Thus, when $v_1' = v_2'$, these two weighted paths have equal lengths and the same endpoints. Because N' has the NELP property, these two weighted paths must then coincide, meaning that P_1' and P_2' have a common suffix of length γ. Moreover, by the definition of v', P_1' and P_2' have no node in common between u' and v'. Therefore $v' = v_1' = v_2'$ implies that $K' = P_1' \cup P_2'$ is a crack with a longest common suffix of length γ, which is the same as the length of the longest common suffix of P_1 and P_2. Because of this, because P_1' and P_2' have a longest common prefix with the same length as the longest common prefix of P_1 and P_2 (α), and finally because P_1' and P_2' are isomorphic to P_1 and P_2, respectively, then K and K' are isomorphic (by Lemma 5).

Now consider the case where K is a closed crack: in this case, P_1 and P_2 only differ for the length assigned to edge (u, v), which must be β_1 in P_1 and β_2 in P_2. Note that P_1' and P_2' cannot separate openly, because otherwise also P_1 and P_2 would separate openly (by Lemma 15). Therefore, P_1' and P_2' must both contain the edge (u', v'), but assign different lengths to it. (We have called the head of this edge v', as it is clearly the first node that P_1' and P_2' have in common after separating at u'.) Because P_1' is isomorphic to P_1, the length assigned to (u', v') in P_1' must be β_1: if this edge were assigned a length $\beta_1' < \beta_1$ in P_1' , then a node at depth $\alpha + \beta_1'$ would exist in P_1' and therefore in P_1, but this contradicts the fact that there is no node in P_1 between u (at depth α) and v (at depth $\alpha + \beta_1$); if instead (u', v') were assigned a length $\beta_1' > \beta_1$ in P_1', then no node would exist at depth $\alpha + \beta_1$ in P_1', which contradicts the fact that v has depth $\alpha + \beta_1$ in P_1. Symmetrically, because P_2' is isomorphic to P_2, the length assigned to (u', v') in P_2' must be β_2. Thus v' has depth $\alpha + \beta_1$ in P_1' and $\alpha + \beta_2$ in P_2', meaning that $v' = v_1' = v_2'$. As we showed above, this implies that $K' = P_1' \cup P_2'$ is a crack isomorphic to K, whenever K is a closed crack.

It remains to prove that for any open crack $K = P_1 \cup P_2$ contained in one of the two networks, there exists a crack K' in the other network that is isomorphic to K. We prove this by induction on the number of edges in the longest suffix common to P_1 and P_2. In both the base case and the inductive step, we let u_1 and u_2 be the direct ancestors of v in P_1 and P_2, respectively. Of these two nodes, at least one is not an ancestor of the other, otherwise either N would contain a cycle or $u_1 = u_2$ (the latter would imply that K is a closed crack). Thus, without loss of generality, we assume throughout that u_2 is not an ancestor of u_1. Note that this implies that $u_2 \neq u$.

Base case. (See Fig. S7 to follow the argument below.) If the longest suffix common to P_1 and P_2 contains no edge, then v coincides with the leaf in P_1 and P_2, and $\gamma = 0$. Thus P_1' and P_2' have lengths $\alpha + \beta_1$ and $\alpha + \beta_2$, respectively, meaning that v_1' and v_2' are their leaves. But P_1' and P_2' have the same leaf, thus implying $v_1' = v_2'$. It remains to prove that $v' = v_1' = v_2'$, or in other words that P_1' and P_2' have no node in common between u' and $v_1' = v_2'$. Because u_2 has outdegree 2 or more, we can define a root-leaf path R in N that separates openly from P_2 at u_2: let R have a common prefix with P_2 consisting of the portion of P_2 from the root down to u_2; then, let R take an edge (u_2, w) with $w \neq v$ (with any of this edge's lengths in N) and finally let R take any weighted path from w that does not end up in v (which is possible because N is funnel-free). Note that R and P_2 have no node in common below u_2 — as otherwise R would contain v, which we excluded by construction — meaning that $P_2 \cup R$ is a wishbone.

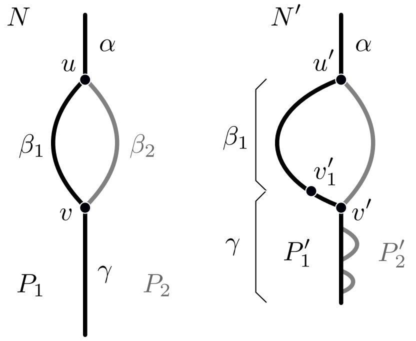

Figure S6. Illustration of the notation for Lemma 18 and of the argument against claim **C1** within it. P_1 and P'_1 are in black and the portions of P_2 and P'_2 not overlapping with P_1 and P'_1 are in grey. Note that the position of v'_2 along P'_2 is not shown as it may be either above or below v'.

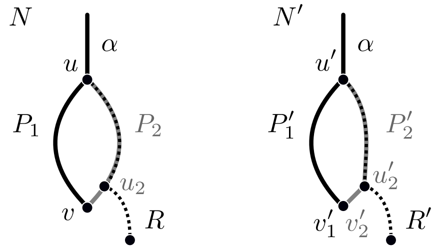

Figure S7. Illustration of the base case of the proof of Lemma 18. P_1 and P'_1 are in black and the portions of P_2 and P'_2 not overlapping with P_1 and P'_1 are in grey. Finally, the portions of R and R' not overlapping with P_1 and P'_1 are represented by the dashed lines.

Moreover, $P_1 \cup R$ is also a wishbone: assuming otherwise would mean that R has a node in common with P_1 below u, either contradicting the fact that P_1 and P_2 have no nodes in common between u and v, or contradicting the requirement that u_2 is not an ancestor of u_1, or contradicting the requirement that v does not belong to R. Now let $W_1 = P_1 \cup R$ and $W_2 = P_2 \cup R$. Because these are wishbones, by Lemma 17, $W'_1 = P'_1 \cup R'$ and $W'_2 = P'_2 \cup R'$ are also wishbones isomorphic to W_1 and W_2, respectively. Moreover, because the same holds for P_1, P_2 and R in N, the following holds:

$$\mu(P'_1, P'_2) = \mu(P'_1, R') = \alpha \leq \mu(P'_2, R'),$$

meaning that P'_1 and R' separate at the same node, u', where P'_1 and P'_2 separate.

Now recall that v' is the first node that P'_1 and P'_2 have in common after separating at u'. Note that v' cannot be in the prefix common to P'_2 and R', because otherwise v' would be a node common to P'_1 and R', which (together with the fact that v' is a strict descendant of u') contradicts the fact that P'_1 and R' form a wishbone and separate at u'. Thus v' must belong to the suffix of P'_2 after separation from R', or, in other words, v' must have a depth in P'_2 strictly greater than $\mu(P'_2, R')$. Now note that the only node in P_2 at a depth strictly greater than $\mu(P_2, R)$ is v, meaning (as P'_2 is isomorphic to P_2) that there can only be one node in P'_2 at a depth strictly greater than $\mu(P'_2, R') = \mu(P_2, R)$. This node is $v'_1 = v'_2$, thus allowing to conclude that $v' = v'_1 = v'_2$.

Inductive case. Now suppose that the longest suffix common to P_1 and P_2 contains $k > 0$ edges, and

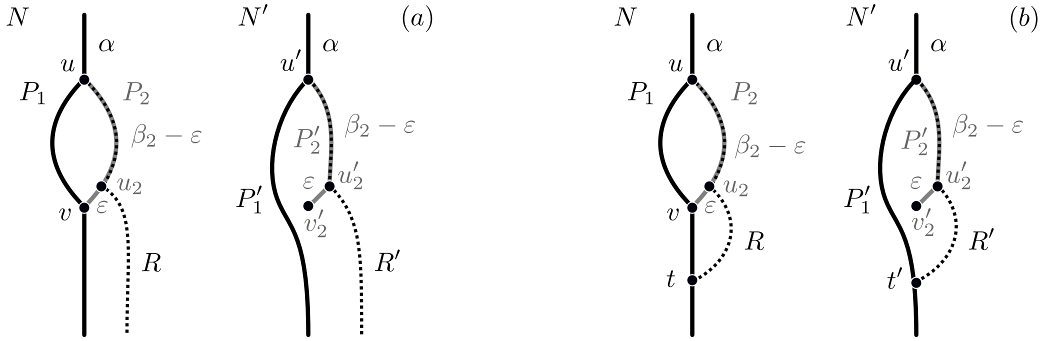

Figure S8. Illustration of the argument against claim **C3** in the proof of Lemma 18. P_1 and P_1' are in black and the portions of P_2 and P_2' not overlapping with P_1 and P_1' are in grey. Finally, the portions of R and R' not overlapping with P_1 and P_1' are represented by the dashed lines. In (a), P_1 and R — and thus P_1' and R' — form a wishbone, whereas in (b), P_1 and R — and thus P_1' and R' — form an open crack.

assume that for every open crack with up to $k-1$ edges in its longest common suffix in one of the two networks, there exists an isomorphic crack in the other network. We show that each of the following claims leads to contradiction: (**C1**) v' is a strict descendant of v_1' along P_1'; (**C2**) v' is a strict descendant of v_2' along P_2'; (**C3**) v' is a strict ancestor of v_2' along P_2'; (**C4**) $v' = v_2'$ and v' is a strict ancestor of v_1' along P_1'.

First, let us deal with claim **C1**: suppose that v' is a strict descendant of v_1' along P_1' (see Fig. S6). We show that for every wishbone or crack W' contained in $P_1' \cup P_2'$ and containing P_1', there exists in N a wishbone or crack W isomorphic to W'. This is trivial when W' is a wishbone: because P_1' and P_2' are root-leaf paths to the same taxon, the only wishbone W' contained in $P_1' \cup P_2'$ and containing P_1' is P_1' itself, for which $W = P_1$. This is also trivial when W' is a closed crack, as we have already proved that N and N' must have the same closed cracks (up to isomorphism). It remains the case where W' is an open crack. In this case, W' can be obtained by combining P_1' with a weighted path contained in P_2' that has no node or edge in common with P_1', other than its endpoints w and z. Because the first such weighted path in P_2' is the one between $w = u'$ and $z = v'$, it follows that z must be a descendant of v' and thus a strict descendant of v_1' along P_1'. Now note that, because P_1' is isomorphic to P_1, and because the suffix of P_1 starting in v contains k edges, then the suffix of P_1' starting in v_1' (the node at the same depth in P_1' as v in P_1) also contains exactly k edges. But then, because z is a strict descendant of v_1' along P_1', then the suffix of P_1' starting in z contains strictly less than k edges. That is, the longest suffix common to the two root-leaf paths composing the open crack W' contains strictly less than k edges. Then, by inductive hypothesis, there must be a crack W in N that is isomorphic to W'. We have thus proved that for every wishbone or crack W' contained in $P_1' \cup P_2'$ and containing P_1', there exists in N a wishbone or crack W isomorphic to W'. This, together with the fact that N satisfies the NELP property, allows us to apply Lemma 10 and conclude that the unique root-leaf paths isomorphic to P_1' and P_2' in N, that is P_1 and P_2, must intersect each other at the same depths as P_1' and P_2'. But this leads to a contradiction, as the node at depth $\alpha + \beta_1$ in P_1, that is v, belongs to both P_1 and P_2, whereas the node at the same depth in P_1', that is v_1' only belongs to P_1'. Similarly, one can prove that claim **C2** leads to contradiction.

Now assume (claim **C3**) that v' is a strict ancestor of v_2' along P_2'. Recall that u_1 and u_2 are the two direct ancestors of v in P_1 and P_2, respectively, with u_2 assumed to not be an ancestor of u_1. Because u_2 has outdegree 2 or more, we can define a root-leaf path R in N that separates openly from P_2 at u_2: let R have a common prefix with P_2 consisting of the prefix of P_2 from the root down to u_2; then, let R take an edge (u_2, w) with $w \neq v$ (with any of this edge's lengths in N) and then let R continue taking

edges always avoiding ending up in v (recall that N is funnel-free), until it either arrives in a leaf or in a node t belonging to $P_1 \cup P_2$. Note that, if such t exists, then it must be a strict descendant of v in the suffix common to P_1 and P_2. (By construction $t \neq v$; moreover, if t belonged to P_2 and not to P_1, then t would lie between u_2 and v in P_2, which contradicts the assumption that u_2 is a direct ancestor of v in P_2; finally if t belonged to P_1 and not to P_2, then t would be an ancestor of u_1, implying that u_2 is an ancestor of u_1, which is not possible by construction.) Finally, from t onwards, let R coincide with the suffix common to P_1 and P_2. (See Fig. S8 to follow the argument below.)

Now consider $P_1 \cup R$. Because $P_1 \cup P_2$ is a crack and u_2 is strictly between u and v, R cannot have any node in common with P_1 between u and u_2 (u_2 included). Thus, if R arrives in a leaf without hitting a node in $P_1 \cup P_2$, then $P_1 \cup R$ is a wishbone. Then, by Lemma 17, $P'_1 \cup R'$ is a wishbone isomorphic to $P_1 \cup R$ (see Fig. S8(a)). If instead R hits $P_1 \cup P_2$ in t, then $P_1 \cup R$ is an open crack: it is a crack because, by construction, t is the first node that R has in common with P_1 after separating from it at u, and because R has its suffix following t in common with P_1; it is open, because $u_2 \neq u$ implies that the path from u to t in R is composed by more than one edge. Moreover the suffix of P_1 and R following t contains fewer than k edges, as t is a strict descendant of v. Thus, by inductive hypothesis, in N' there exists a crack isomorphic to $P_1 \cup R$. Because isomorphic cracks are the union of isomorphic root-leaf paths (Lemma 5) and because in N' there can be no root-leaf path isomorphic to P_1 and R other than P'_1 and R', respectively (as N' satisfies the NELP property, by Lemma 9), then this crack can be written as $P'_1 \cup R'$ (see Fig. S8(b)). Thus, we have proved that $P'_1 \cup R'$ is either a wishbone or a crack isomorphic to $P_1 \cup R$. Moreover, because the same holds for P_1, P_2 and R in N, the following holds:

$$\mu(P'_1, P'_2) = \mu(P'_1, R') = \alpha \leq \mu(P'_2, R') = \alpha + \beta_2 - \varepsilon,$$

where ε denotes the length of edge (u_2, v) in P_2. Thus P'_2 and R' separate at a node that we denote by u'_2, which has depth $\alpha + \beta_2 - \varepsilon$ in P'_2 (the same as u_2 in P_2). When $P'_1 \cup R'$ is a crack, because it is isomorphic to $P_1 \cup R$, the first node in the suffix common to P'_1 and R' must be at the same depths in P'_1 and R' as t in P_1 and R. We call this node t'. Because the depth of t in R is strictly larger than $\alpha + \beta_2 - \varepsilon$, the same holds for the depth of t' in R', implying that t' must be a strict descendant of u'_2.

Now recall that v' is the first node that P'_1 and P'_2 have in common after separating at u'. There are two possibilities regarding its position in P'_2 relative to u'_2. First consider the case where v' is an ancestor of u'_2 along P'_2 (including $v' = u'_2$). In this case v' is a strict descendant of u' in the prefix common to P'_2 and R', and at the same time v' is a node in P'_1. But this contradicts both possible relations between P'_1 and R': P'_1 and R' forming a wishbone and separating at u', and P'_1 and R' forming a crack by separating at u' and joining in t' (which, as we showed, must be a strict descendant of u'_2, implying $t' \neq v'$). The other case to consider is that of v' being a strict descendant of u'_2 along P'_2, while being a strict ancestor of v'_2 along P'_2 (claim **C3**). That is, v' is strictly between u'_2 and v'_2 in P'_2. But this is impossible, as together with the fact that P_2 and P'_2 are isomorphic, it would imply the existence of a node strictly between u_2 and v in P_2, which is excluded by construction. Since all these possibilities lead to a contradiction, we conclude that **C3** is also impossible.

Finally, let us deal with claim **C4**: suppose that $v' = v'_2$, and that v' is a strict ancestor of v'_1 along P'_1. (See Fig. S9 to follow the argument below.) Recall that, because P'_1 and P'_2 are isomorphic to P_1 and P_2, they have lengths $\alpha + \beta_1 + \gamma$ and $\alpha + \beta_2 + \gamma$, respectively. Also recall that P'_1 and P'_2 separate at u' (with depth α in P'_1 and P'_2) and that the respective depths of v'_1 and $v' = v'_2$ in P'_1 and P'_2 are $\alpha + \beta_1$ and $\alpha + \beta_2$. Now, let $\delta > 0$ denote the length of the weighted path in P'_1 from v' to v'_1, implying that the weighted path in P'_1 from u' to v' has length $\beta_1 - \delta > 0$ (strictly positive because by construction $u' \neq v'$). Now define in N' a new root-leaf path S' that coincides with P'_1 and P'_2 along all their common prefix down to u' (of length α), then coincides with P'_1 along the weighted path in P'_1 from u' to v' (of length $\beta_1 - \delta$), and finally coincides with P'_2 along all its suffix from v' onwards (of length γ). As a result, S' and P'_1 have a common prefix down to v' and therefore $\mu(P'_1, S') \geq \alpha + \beta_1 - \delta$.

Let us now focus on the consequences on N of these definitions. Let S denote the unique root-leaf path in N isomorphic to S' (Lemma 16) and let s be the node at depth $\alpha + \beta_1 - \delta$ in P_1 (whose existence

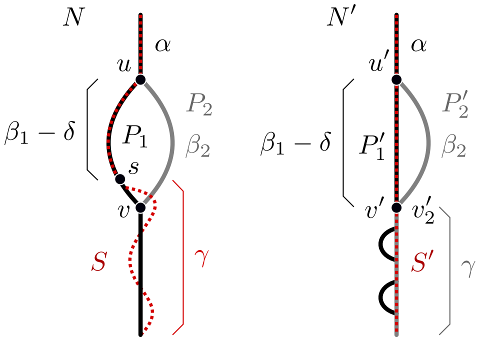

Figure S9. Illustration of the argument against claim **C4** in the proof of Lemma 18. P_1 and P'_1 are in black and the portions of P_2 and P'_2 not overlapping with P_1 and P'_1 are in grey. Finally S and S' are represented by the dashed lines in red.

is guaranteed by the existence of node v' at the same depth in P'_1). Because the same holds for P'_1, P'_2 and S' in N', the following holds (Lemma 15):

$$\mu(P_1, P_2) = \mu(P_2, S) = \alpha < \alpha + \beta_1 - \delta \leq \mu(P_1, S),$$

meaning that the prefix of P_1 down to s is entirely in common with S (and therefore S and P_2 separate in u). But this implies that all the nodes that S has in common with P_2 after separating from it at u must be strict descendants of s, as otherwise P_1 and P_2 would have nodes in common between u and v, which is excluded by construction.

Now note that, because P'_2 is isomorphic to P_2, and because the suffix of P_2 starting in v contains k edges, then the suffix of P'_2 starting in $v' = v'_2$ (the node at the same depth in P'_2 as v in P_2) also contains exactly k edges. But this suffix coincides with that of S' starting in $v' = v'_2$. Then, also the suffix of S starting in s (the node at the same depth in S as v' in S') contains exactly k edges. This observation, together with the fact that all the nodes that S has in common with P_2 after separating from it must be strict descendants of s, allows us to show that for every wishbone or crack W contained in $P_2 \cup S$ and containing S, there exists in N' a wishbone or crack W' isomorphic to W. This is trivial when W is a wishbone: because P_2 and S are root-leaf paths to the same taxon, the only wishbone W contained in $P_2 \cup S$ and containing S is S itself, for which $W' = S'$. This is also trivial when W is a closed crack, as we have already proved that N and N' must have the same closed cracks (up to isomorphism). It remains the case where W is an open crack. In this case, it can be written as $W = S \cup Q$, where Q is a root-leaf path contained in $P_2 \cup S$, separating openly from S at some internal node in S. Because all the nodes that S and P_2 have in common after separating at u must be strict descendants of s, also the first node in the longest suffix common to S and Q must be a strict descendant of s, meaning that this suffix must contain strictly less than k edges. Then, by inductive hypothesis, there must be a crack W' in N' that is isomorphic to $W = S \cup Q$. Because for every wishbone or crack W contained in $P_2 \cup S$ and containing S, there exists in N' a wishbone or crack W' isomorphic to W, then P'_2 and S' must intersect each other at the same depths as P_2 and S (by Lemma 10). But this leads to a contradiction, as the node at depth $\alpha + \beta_1 - \delta$ in S', that is $v' = v'_2$, belongs to both S' and P'_2, whereas the node at the same depth in S, that is s, only belongs to S.

We have thus proved that each of **C1-C4** leads to a contradiction. The fact that neither **C2** nor **C3** can hold implies that $v' = v'_2$. This, together with the fact that neither **C1** nor **C4** can hold, implies $v' = v'_1$. Thus, $v' = v'_1 = v'_2$, which, as explained in the introduction of this proof, implies that $P'_1 \cup P'_2$ is a crack isomorphic to $P_1 \cup P_2$, and thus the lemma follows. □

Proposition 2 follows from Corollary 4 and Lemma 18.

Networks with inheritance probabilities and likelihood-based reconstruction

As described in the main text, the ML framework we consider [2, 32, 33, 38] not only models edge lengths, but also inheritance probabilities. The latter provide, for each reticulate edge in a network N, the probability that a random tree T displayed by N includes that edge (i.e., that inheritance follows that edge). The inheritance probabilities determine, for each tree $T \in \mathcal{T}(N)$, an associated probability $\mathbf{Pr}(T|N)$.

Unfortunately, including inheritance probabilities does not solve identifiability problems: in Fig. S10 we show an example of two phylogenetic networks N_1, N_2 with edge lengths and inheritance probabilities that cannot be distinguished on the basis of the trees they display and associated probabilities. By setting the edge lengths as shown, and the inheritance probabilities so that $p_2 = 1-(1-p_1)(1-q_1)$ and $q_2 = p_1/p_2$, it is easy to check that $\mathbf{Pr}(T_1|N_1) = \mathbf{Pr}(T_1|N_2)$, $\mathbf{Pr}(T_2|N_1) = \mathbf{Pr}(T_2|N_2)$ and $\mathbf{Pr}(T_3|N_1) = \mathbf{Pr}(T_3|N_2)$. Thus, for every assignment of edge lengths and inheritance probabilities to N_1, there exist corresponding assignments to N_2 that make the resulting networks display the same trees, with the same edge lengths and the same probabilities of being observed. Because $\mathcal{T}(N_1) = \mathcal{T}(N_2)$, and $\mathbf{Pr}(T|N_1) = \mathbf{Pr}(T|N_2)$ for any T displayed by these two networks, it is easy to see that

$$\prod_{i=1}^{m} \sum_{T \in \mathcal{T}(N_k)} \mathbf{Pr}(A_i|T)\mathbf{Pr}(T|N_k).$$

is the same for $k = 1$ or $k = 2$, that is, the likelihoods of N_1 and N_2 are identical regardless of the data. In other words, no method based on this definition of likelihood will be able to discriminate between them.

The example above is not an exception: for any two distinct indistinguishable networks (i.e., with $\mathcal{T}(N_1) = \mathcal{T}(N_2)$), it is possible to provide assignments of inheritance probabilities to their reticulations, so that not only these networks display the same trees, but that also the probabilities associated to the trees they display are identical.

Moreover, we can extend the notion of indistinguishability, as well as that of canonical form, to networks with inheritance probabilities. Results entirely analogous to those we presented in this paper will then hold. We will not prove any of this here, as it lies beyond the scope of the present study.

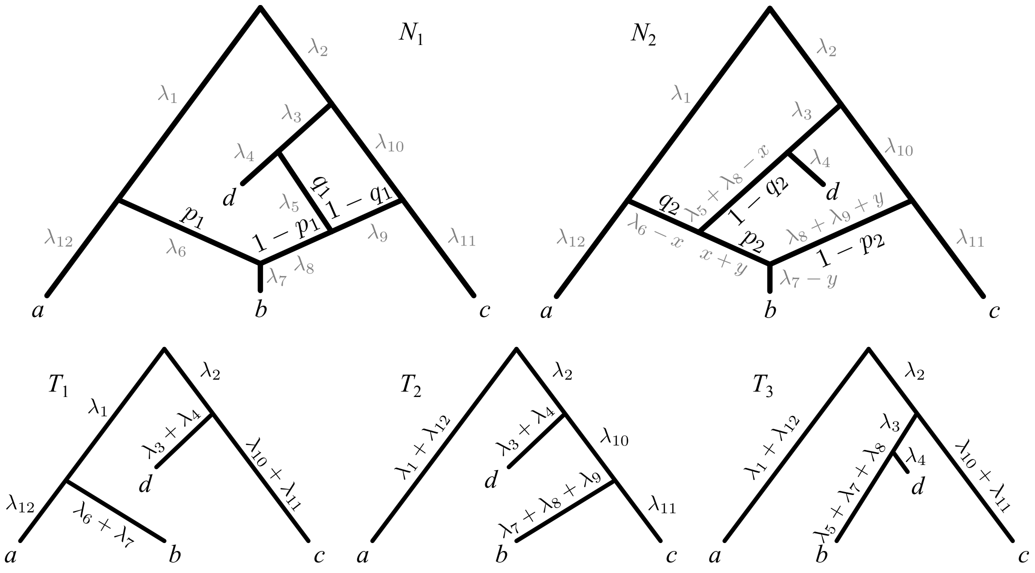

$\mathbf{Pr}(T_1|N_1) = p_1$ $\mathbf{Pr}(T_2|N_1) = (1 - p_1)(1 - q_1)$ $\mathbf{Pr}(T_3|N_1) = (1 - p_1)q_1$

$\mathbf{Pr}(T_1|N_2) = p_2q_2$ $\mathbf{Pr}(T_2|N_2) = 1 - p_2$ $\mathbf{Pr}(T_3|N_2) = p_2(1 - q_2)$

Figure S10. An example showing that the ML framework considered in the main text is also subject to identifiability problems: the two networks N_1 and N_2 (*top*) with edge lengths (gray) and inheritance probabilities (black), display the same trees T_1, T_2, T_3 (*middle*), with the same associated probabilities (*bottom*), when $p_2 = 1 - (1 - p_1)(1 - q_1)$ and $q_2 = p_1/p_2$.
