# Supplementary material for: Correction: Reconstructible Phylogenetic Networks: Do Not Distinguish the Indistinguishable
Source: PLoS Comput Biol. 2019 Jun 14;15(6):e1007137. doi: 10.1371/journal.pcbi.1007137 (PMC6568377; doi:10.1371/journal.pcbi.1007137)
Supplement: S1 File — (PDF) [file pcbi.1007137.s001.pdf]

PLOS | COMPUTATIONAL BIOLOGY

RESEARCH ARTICLE

Reconstructible Phylogenetic Networks: Do Not Distinguish the Indistinguishable

Fabio Pardi[1,3]*, **Celine Scornavacca**[2,3]

1 Laboratoire d'Informatique, de Robotique et de Microélectronique de Montpellier (LIRMM, UMR 5506) CNRS, Université de Montpellier, France, **2** Institut des Sciences de l'Evolution de Montpellier (ISE-M, UMR 5554) CNRS, IRD, Université de Montpellier, France, **3** Institut de Biologie Computationnelle, Montpellier, France

* fabio.pardi@lirmm.fr

OPEN ACCESS

Citation: Pardi F, Scornavacca C (2015) Reconstructible Phylogenetic Networks: Do Not Distinguish the Indistinguishable. PLoS Comput Biol 11(4): e1004135. doi:10.1371/journal.pcbi.1004135

Editor: Barbara R Holland, University of Tasmania, Australia

Received: October 25, 2014

Accepted: January 19, 2015

Published: April 7, 2015

Copyright: © 2015 Pardi, Scornavacca. This is an open access article distributed under the terms of the Creative Commons Attribution License, which permits unrestricted use, distribution, and reproduction in any medium, provided the original author and source are credited.

Data Availability Statement: All relevant data are within the paper and its Supporting Information files.

Funding: This work was funded by a PEPS BMI 2013 grant (http://www.cnrs.fr/mi/spip.php?article315) from the Centre Nationale de la Recherche Scientifique. This publication is contribution no. 2015-011 of the Institut des Sciences de l'Evolution de Montpellier (ISEM, UMR 5554). The funders had no role in study design, data collection and analysis, decision to publish, or preparation of the manuscript.

Competing Interests: The authors have declared that no competing interests exist.

Abstract

Phylogenetic networks represent the evolution of organisms that have undergone reticulate events, such as recombination, hybrid speciation or lateral gene transfer. An important way to interpret a phylogenetic network is in terms of the trees it displays, which represent all the possible histories of the characters carried by the organisms in the network. Interestingly, however, different networks may display exactly the same set of trees, an observation that poses a problem for network reconstruction: from the perspective of many inference methods such networks are indistinguishable. This is true for all methods that evaluate a phylogenetic network solely on the basis of how well the displayed trees fit the available data, including all methods based on input data consisting of clades, triples, quartets, or trees with any number of taxa, and also sequence-based approaches such as popular formalisations of maximum parsimony and maximum likelihood for networks. This identifiability problem is partially solved by accounting for branch lengths, although this merely reduces the frequency of the problem. Here we propose that network inference methods should only attempt to reconstruct what they can uniquely identify. To this end, we introduce a novel definition of what constitutes a uniquely reconstructible network. For any given set of indistinguishable networks, we define a canonical network that, under mild assumptions, is unique and thus representative of the entire set. Given data that underwent reticulate evolution, only the canonical form of the underlying phylogenetic network can be uniquely reconstructed. While on the methodological side this will imply a drastic reduction of the solution space in network inference, for the study of reticulate evolution this is a fundamental limitation that will require an important change of perspective when interpreting phylogenetic networks.

Author Summary

We consider here an elementary question for the inference of phylogenetic networks: what networks can be reconstructed. Indeed, whereas in theory it is always possible to reconstruct a phylogenetic tree, given sufficient data for this task, the same does not hold for

phylogenetic networks: most notably, the relative order of consecutive reticulate events cannot be determined by standard network inference methods. This problem has been described before, but no solutions to deal with it have been put forward. Here we propose limiting the space of reconstructible phylogenetic networks to what we call "canonical networks". We formally prove that each network has a (usually unique) canonical form—where a number of nodes and branches are merged—representing all that can be uniquely reconstructed about the original network. Once a canonical network $\hat{N}$ is inferred, it must be kept in mind that—even with perfect and unlimited data—the true phylogenetic network is just one of the potentially many networks having $\hat{N}$ as canonical form. This is an important difference to what biologists are used to for phylogenetic trees, where in principle it is always possible to resolve uncertainties, given enough data.

Introduction

Explicit [1] or *evolutionary* [2, 3] phylogenetic networks are used to represent the evolution of organisms or genes that may inherit genetic material from more than one source. This may be caused by events such as hybrid speciation (e.g. in plants and animals [4, 5]), horizontal gene transfer (e.g. in bacteria [6, 7]), viral reassortment [8], or recombination (e.g. in viruses [9, 10] or in the genomes of sexually reproducing species [11–13]). They are called "explicit" to distinguish them from "implicit" [14], "abstract" [1] or "data-display" [3] phylogenetic networks, which are used to display collections of alternative evolutionary hypotheses supported by conflicting signals in the data. In explicit networks, multiple-inheritance events are represented as *reticulations*, that is, nodes where two or more lineages converge to give rise to a new lineage, whose genetic material is a combination of that of its direct ancestors.

Explicit networks can be interpreted in terms of classic, tree-like evolution: if we focus on a single, indivisible and thus non-recombining inherited character (for example a single site in a DNA sequence), its history is still best described by a tree. This observation gives rise to the notion of *trees displayed by a network*, which are all the possible single-character histories implied by a phylogenetic network. (See, e.g., Fig. 1, where T_1, T_2 and T_3 are the trees displayed by networks N_1 and N_2. Formal definitions are in the Results section.)

Several works in the last few years have focused on the methodology for phylogenetic network inference, and data-display networks in particular have begun to make a real impact on the everyday practice of biologists (e.g., [15–17]). There remains, however, a strong demand for automatic reconstruction of networks that not only display conflicting signals in the data, but also seek to explain these signals with explicit inferences of past reticulation events (see, e.g., [18–20]). This is evidenced, for example, by the abundance of manually reconstructed networks in the literature [8, 21–27]. As a result of this demand, the inference of explicit networks is now a rapidly growing field of research [1].

Some paradigms in the proposed methodology are beginning to emerge. Not surprisingly, the notion of trees displayed by a phylogenetic network plays a central role: the general idea is to evaluate the fit of a network N with the data *indirectly*—on the basis of how well the trees displayed by N explain the data. In the following, we describe how this applies to the two main approaches for explicit network reconstruction: consistency-based approaches (see [28] for a survey)—seeking a network consistent with a number of prior evolutionary inferences (typically trees or groupings of taxa)—and sequence-based approaches, such as standard formulations of maximum parsimony and maximum likelihood for networks [2, 29–33].

Although evaluating a network via the trees it displays is evolutionarily meaningful, it has a problematic consequence: from the perspective of these reconstruction methods, all networks displaying the same set of trees are "indistinguishable", as the function that these methods seek to optimize will always assign the same score to all networks displaying the same set of trees, regardless of the input data. In other words, the central parameter of phylogenetic network inference, the network itself, is in some cases not identifiable.

An Identifiability Problem

As an example, consider again networks N_1 and N_2 in Fig. 1, which display the same trees $\mathcal{T}(N) = \{T_1, T_2, T_3\}$. (In the following, $\mathcal{T}(N)$ denotes the set of trees displayed by N.) By displaying the same trees, these networks display the same clades, the same triples, the same quartets (triples and quartets are rooted subtrees with 3 leaves and unrooted subtrees with 4 leaves, respectively) and in general the same subtrees with an arbitrary number of leaves. Therefore, any method that reconstructs a network based on its consistency with collections of such data will not be able to distinguish between networks N_1 and N_2. This includes all the methods whose data consists of clusters of taxa (e.g., [34]), triples (e.g., [35]), quartets (e.g., [36]), or any trees (e.g., [37]).

The same holds for many, sequence-based, maximum parsimony and maximum likelihood approaches proposed in recent papers. For maximum parsimony, a practical approach [2, 29–31] is to consider that the input is partitioned in a number of alignments $A_1, A_2, \ldots, A_m$, each from a different non-recombining genomic region (possibly consisting of just one site each), and then take, for each of these alignments, the best parsimony score $\mathbf{Ps}(T|A_i)$ among all those of the trees displayed by a network N. The parsimony score of N is then the sum of all the parsimony scores thus obtained. Formally, we have

$$\mathbf{Ps}(N|A_1, A_2, \ldots, A_m) = \sum_{i=1}^{m} \min_{T \in \mathcal{T}(N)} \mathbf{Ps}(T|A_i).$$

It is clear that if two networks display the same set of trees (as in Fig. 1), then their parsimony score with respect to any input alignments will be the same—because they take the minimum

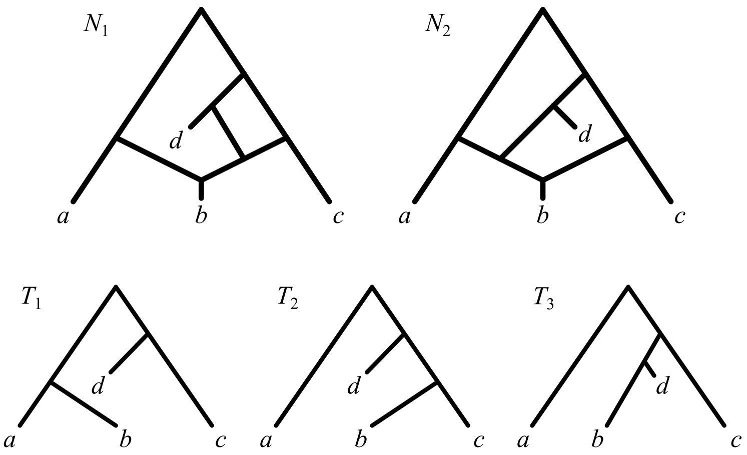

Fig 1. Indistinguishable network topologies. The network topologies N_1 and N_2 are indistinguishable to most current approaches for network reconstruction, as they display the same tree topologies T_1, T_2 and T_3.

doi:10.1371/journal.pcbi.1004135.g001

value over the same set $\mathcal{T}(N)$—and thus they are indistinguishable to any method based on the maximum parsimony principle above.

As for maximum likelihood (ML), Nakhleh and collaborators [2, 32, 33, 38] have proposed an elegant framework whereby a phylogenetic network N is not only described by a network topology, but also edge lengths and inheritance probabilities associated to the reticulations of N. As a result, any tree T displayed by N has edge lengths—allowing the calculation of its likelihood $\mathbf{Pr}(A|T)$ with respect to any alignment A—and an associated probability of being observed $\mathbf{Pr}(T|N)$. The likelihood function with respect to a set of alignments $A_1, A_2, \ldots, A_m$, each from a different non-recombining genomic region, is then given by:

$$\mathbf{Pr}(A_1, A_2, \ldots, A_m|N) = \prod_{i=1}^{m} \mathbf{Pr}(A_i|N) = \prod_{i=1}^{m} \sum_{T \in \mathcal{T}(N)} \mathbf{Pr}(A_i|T)\ \mathbf{Pr}(T|N).$$

Note that an important difference with the consistency-based and parsimony methods described above is that any tree T displayed by a network has now edge lengths and an associated probability $\mathbf{Pr}(T|N)$.

Unfortunately, this ML framework is also subject to identifiability problems. For example, it does not allow us to distinguish between networks with topologies N_1 and N_2 in Fig. 1: for every assignment of edge lengths and inheritance probabilities to N_1, there exist corresponding assignments to N_2 that make the resulting networks indistinguishable, that is, displaying the same trees, with the same edge lengths and the same probabilities of being observed (see the last section in the Supporting Information, S1 Text). As a result, the likelihoods of these two networks will be identical, regardless of the data, and no method based on this definition of likelihood will be able to favour one of them over the other. We refer to S1 Text for a more detailed discussion about networks with inheritance probabilities and likelihood-based reconstruction.

In general, we believe that these identifiability problems affect all network inference methods which seek consistency with unordered collections of sequence alignments or pre-inferred attributes such as clusters, triples, quartets or trees.

The Importance of Edge Lengths

In this paper, as in the ML framework above, we adopt networks and trees with edge lengths as the primary objects of our study. The primary motivation for this is that this choice makes our results directly relevant to the statistical approaches for network inference, all of which need edge lengths to measure the fit of a phylogeny with the available data. In addition to ML, these approaches include distance-based and Bayesian methods [39], which are also promising for future work.

However, there is another motivation for our choice: accounting for edge lengths solves some of the identifiability problems outlined above, as in some cases it allows to distinguish between networks with different topologies, which would be otherwise impossible to tell apart. For example, consider the three network topologies in Fig. 2 (top), where taxon o is an outgroup used to identify the root of the phylogeny for a, b and c. These networks show three very different evolutionary histories: in N_1 taxon b is the only one issued of a reticulation event—in other words the genome of b is recombinant—whereas in N_2 and N_3, it is a and c, respectively, that are recombinant. However, N_1, N_2 and N_3 display the same tree topologies—those of T_1 and T_2—and thus would be indistinguishable to any approach that does not model edge lengths.

If instead edge lengths are accounted for (e.g. in a ML context) and the data supports T_1 and T_2 with the edge lengths in Fig. 2, then the only network fitting perfectly the data is N_2, with

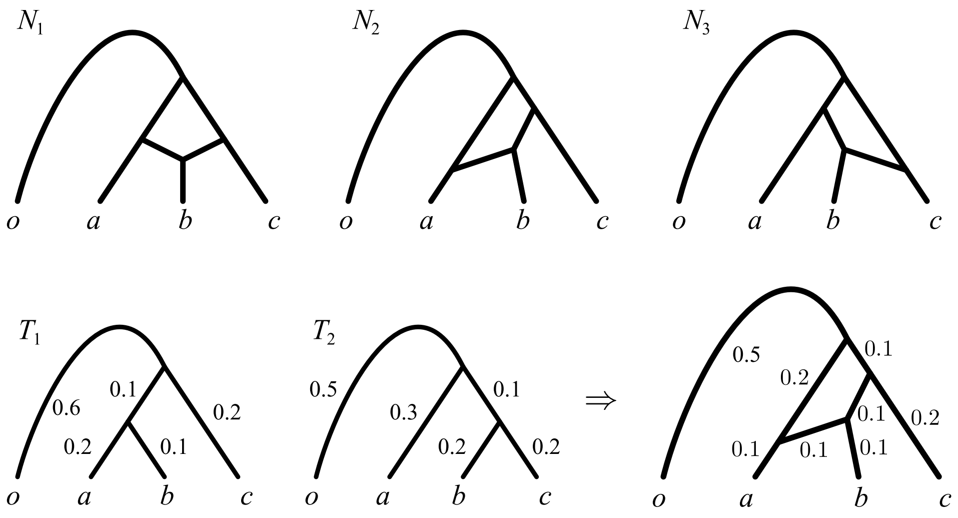

Fig 2. Edge lengths are informative to distinguish among different network topologies. The only network topology, among N_1, N_2 and N_3 that can display simultaneously T_1 and T_2 with the indicated edge lengths is N_2: see for example the edge lengths assignment in the bottom right corner.

doi:10.1371/journal.pcbi.1004135.g002

the edge lengths in Fig. 2 (bottom right). It is easy to check that N_2 now displays T_1 and T_2 with the shown edge lengths, whereas no edge length assignment to N_1 or N_3 can make these networks display T_1 and T_2.

We note that, throughout this paper, as in classical likelihood approaches, edge lengths measure evolutionary divergence, for example in terms of expected number of substitutions per site. No molecular clock is assumed, meaning that we do not expect edge lengths to be proportional to time.

Remaining Identifiability Problems, and a Proposed Solution

Unfortunately, accounting for edge lengths only solves some of the identifiability problems for phylogenetic networks. Consider networks N_1 and N_2 in Fig. 3: for any set of edge lengths for N_1, there exist an infinity of edge length assignments for N_2 that make these two networks display exactly the same set of trees with the same edge lengths. In the following, we say that networks such as N_1 and N_2 are *indistinguishable*.

In fact it is not difficult to construct other examples of indistinguishable networks: each time a network has a reticulation v giving birth to only one edge (i.e. with outdegree 1), then we can reduce by $\Delta\lambda$ the length of this edge and correspondingly increase by $\Delta\lambda$ the lengths of the edges ending in v, without altering the set of trees displayed by the network. Note that this operation, which we refer to as "unzipping" reticulation v, can result in v coinciding with a speciation node or a leaf when $\Delta\lambda$ is taken to equal the length of the edge going out of v. For example in Fig. 3, one may fully unzip the two reticulation nodes in N_1, thus obtaining the network N' of Fig. 4. As expected, N_1 and N' display the same set of trees ($\{T_1, T_2, T_3\}$) and are thus indistinguishable. What is most interesting in this example is that, if we fully unzip the two reticulations in N_2 (the other network in Fig. 3, also displaying $\{T_1, T_2, T_3\}$), then we eventually end up obtaining N' again. As we shall see in the following, this is not a coincidence: the unzipping transformations described above lead to what we call the *canonical form* of a network; under mild assumptions, two networks are indistinguishable if and only if they have the same

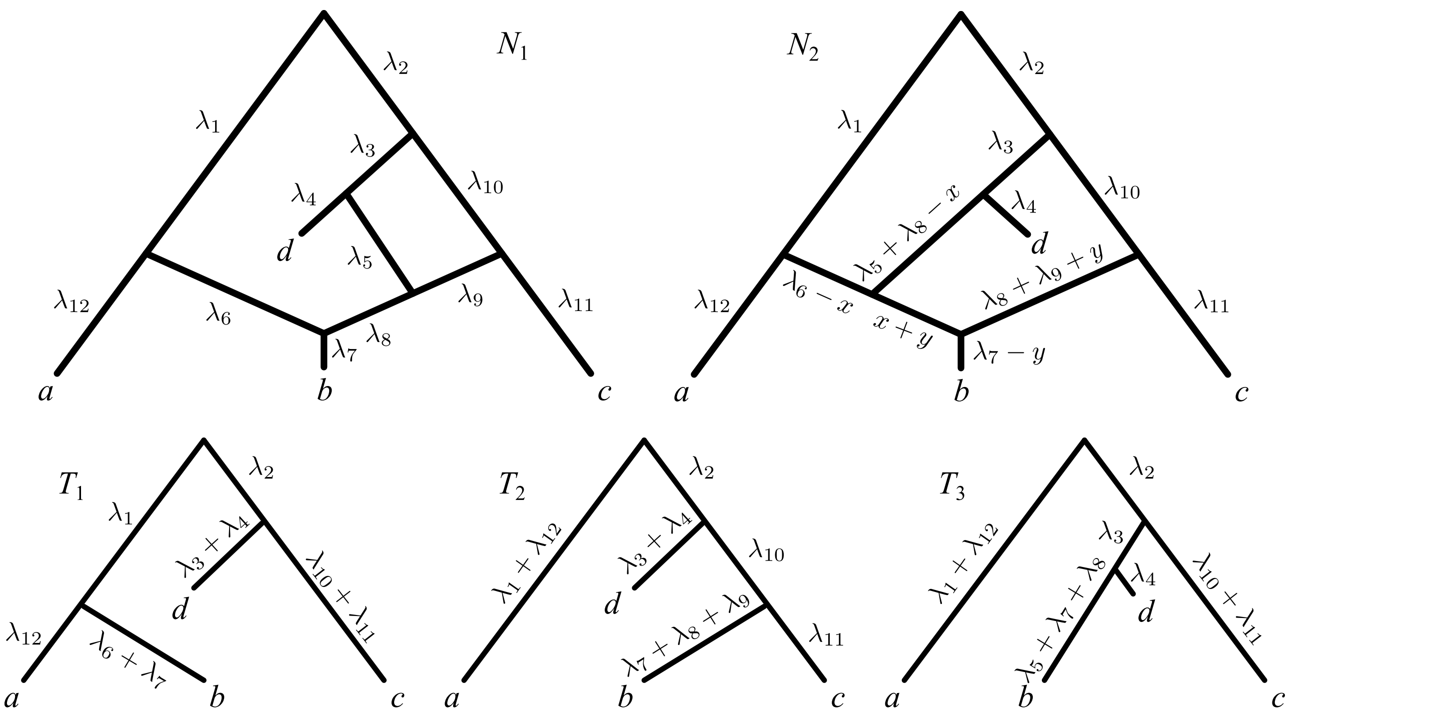

Fig 3. Two networks with edge lengths N_1, N_2 displaying the same set of trees $\mathcal{T}(N_1) = \mathcal{T}(N_2) = \{T_1, T_2, T_3\}$. For any choice of edge lengths $\lambda_1, \lambda_2, \ldots, \lambda_{12}$ for N_1, we define a family of edge length assignments for N_2, parameterized by x, y (with $-y < x < \min\{\lambda_6, \lambda_5 + \lambda_8\}$, $0 < y < \lambda_7$).

doi:10.1371/journal.pcbi.1004135.g003

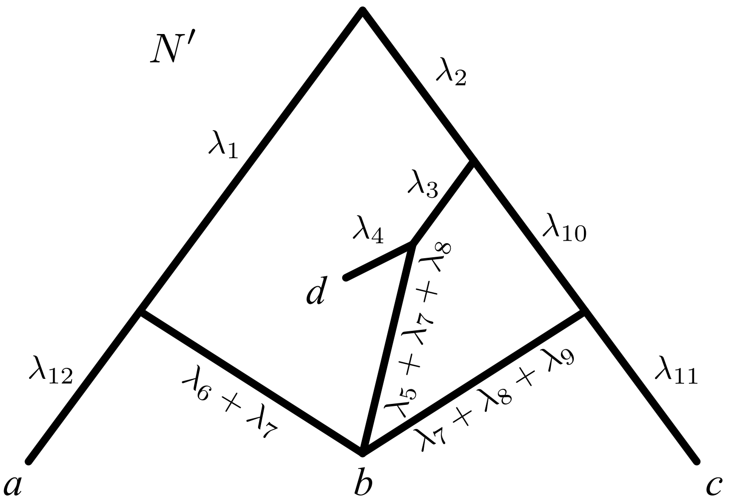

Fig 4. Canonical form of N_1 and N_2 in Fig. 3.

doi:10.1371/journal.pcbi.1004135.g004

canonical form (e.g. N_1, N_2 in Fig. 3 have the same canonical form N'; formal definitions and statements in the Results section).

Here, we propose to deal with the identifiability issues for phylogenetic networks in the following way: since no data will ever enable any of the standard inference methods described above to prefer a network over all of its indistinguishable equivalents, we propose that these methods *should only attempt to reconstruct what they can uniquely identify*, that is, networks in

canonical form. This is a radical shift, not only for the developers of phylogenetic inference methods, who will see a drastic reduction of the solution space of their algorithms, but also for evolutionary biologists, who should abandon their hopes of seeing a network such as N_1 or N_2 in Fig. 3 being reconstructed by these inference methods.

Previous Work and Comparison

Limiting the scope of network reconstruction to topologically-constrained classes of networks has been a recurring theme and an important goal in the literature on phylogenetic networks. Examples of such classes include *galled trees* [40, 41], *galled networks* [42], *level-k networks* [43], *tree-child networks* [44], *tree-sibling networks* [45], networks with *visible reticulations* [1]. Although the ultimate goal should be to establish what can be inferred from biological data, most of the proposed definitions are computationally-motivated: in general the rationale behind these classes is the possibility of devising an efficient algorithm to solve some formalization of the reconstruction problem. None of these definitions claims to have biological significance.

Our goals are more basic: starting from the observation that not all phylogenetic networks are identifiable, since many of them are mutually indistinguishable with most inference approaches, we aim to define a class of networks that is (*existence* goal) large enough that every phylogenetic network has an equivalent (i.e. indistinguishable) network within this class and (*distinguishability* goal) small enough that no two networks within this class are indistinguishable. From our standpoint, the computationally-motivated definitions above are at the same time too broad and too restrictive. Too broad, because they determine a set of networks that includes many pairs of indistinguishable networks: for example the three indistinguishable networks in Fig. 2 are all galled trees—and thus belong to every single one of the classes mentioned above (which are all generalizations of galled trees). Too restrictive, because these classes of networks do not include simple networks that it should be possible to reconstruct from real data. For example, Fig. 5a shows a network N with edge lengths that is not tree-sibling, nor has the visible property, and thus is not galled, nor tree-child (for definitions, see [1]), but which in practice should be reconstructible: apart from the lengths of three edges (x, y, z), N is uniquely determined by the trees that it displays (a consequence of the formal results that we will show in the following), meaning that, given large amounts of data strongly supporting each of these (seven) trees with their correct edge lengths, any method for network inference properly accounting for edge lengths (e.g. based on ML) should be able to reconstruct N, or its canonical form N'.

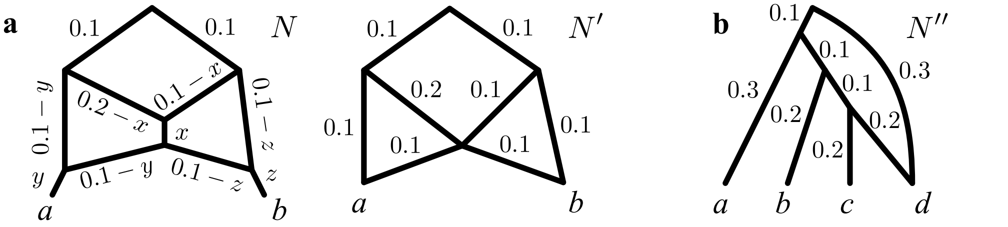

Fig 5. Examples of networks that can be uniquely recovered from the data they generate, despite being excluded by many proposed definitions of reconstructible networks. (*a*) A network N, and its canonical form N', whose topologies are not galled trees, nor galled, tree-child, tree-sibling or regular networks, nor networks with visible reticulations. N, however, is uniquely determined by the trees it displays, with the exception of x, y and z, which can assume any value between 0 and 0.1. Because of the impossibility to determine these values, the canonical form N' has the corresponding edges collapsed. As N' is a network in canonical form satisfying the mild conditions of Corollary 2, N' is uniquely determined by the trees it displays. Note that N provides the biological interpretation for N'. (*b*) The network topology of N'' is such that there exists no regular network displaying the same set of (two) tree topologies as N''. Thus, restricting the scope of phylogenetic inference to regular networks would be very limiting. In our framework, N'' is a network in canonical form and thus uniquely determined by the trees it displays.

doi:10.1371/journal.pcbi.1004135.g005

To the best of our knowledge, only three classes of networks have claims of unique identifiability: *reduced* networks [46, 47], *regular* networks [48] and binary galled trees with no gall containing exactly 4 nodes [49]. These approaches bear some resemblances to ours, but do not include edge lengths in the definition of a network. Moreover, we argue that these classes of networks are still too narrow to be biologically relevant. We briefly describe and comment these previous works below.

Moret et al. [46] defined notions of reconstructible, indistinguishable and reduced networks that resemble concepts that we will introduce here. Although some of their results were flawed [47, 50], some of the arguments in this introduction are inspired by their paper. Particularly relevant to the current paper is a reduction algorithm to transform a network into its *reduced version*. (However, the exact definition of the reduced version is unclear: as one of the authors later pointed out [47], "the reduction procedure of Moret et al. [46] is, in fact, inaccurate" and "in this paper we do not attempt to fix the procedure".) The concept of reduced version is analogous to that of canonical form here, as the authors claim that networks displaying the same tree topologies have the same reduced version (up to isomorphism; Theorem 2 in [46]). This is somehow a weaker analogue of one of our results (Corollary 1); weaker, because it does not claim that, conversely, networks with the same reduced version display the same tree topologies. To have an idea of the difference between our canonical form and the reduced version of Moret and colleagues, in Fig. 6 we compare the canonical form and the reduced version of the same network N_1. (N_1 and its reduced version are taken from Fig. 15 of [46] to avoid possible issues with the reduction algorithm.) As one can see, the canonical form retains more of the complexity of the original network.

Another reduction procedure on network topologies has been studied by Gambette and Huber [49], who prove that if two network topologies reduce to the same topology, then they must display the same tree topologies. Again, this is analogous to, but somehow weaker than our results, since it only provides a sufficient condition for networks to be indistinguishable (which in their context means to display the same tree topologies). This means that there can be irreducible networks that are indistinguishable (e.g. those in Fig. 2) thus failing to achieve the distinguishability goal. Moreover, Gambette and Huber [49] show that a particular class of network topologies (binary galled trees with no gall containing exactly 4 nodes) are uniquely identified by the tree topologies they display. It is clear that this class is too small to achieve the existence goal.

Finally, a regular network is a network topology N in which, among other requirements, no two distinct nodes have the same set of descendant leaves (see [48] for a formal definition and characterizations). This requirement implies, among other things, that N cannot contain any reticulation v with outdegree 1 (v and its direct descendant would have the same descendant leaves), which in turn implies that regular networks are special cases of our canonical networks (the latter however also specify edge lengths). In fact regular networks satisfy a property that is analogous to the one we prove here for canonical networks: a regular network N is uniquely determined by the tree topologies that it displays [51], meaning that there can be no other regular network N' displaying exactly the same set of tree topologies. Willson [51] shows this constructively by providing an algorithm that, given the (exponentially large) set of tree topologies displayed by a regular network R, reconstructs R itself. However, unlike for our canonical forms, for a given network there may exist no regular network displaying the same set of trees (e.g. consider the topology of N'' in Fig. 5b), thus failing to meet the existence goal. Regularity is in fact a very restrictive constraint for a network. For example, none of the networks in Fig. 5 and Fig. 7 is regular, despite the fact that their topologies are uniquely determined by the trees with edge lengths that they display (a consequence of our results further below). Finally, going back to Fig. 6, collapsing the edge above taxa c and d in $R(N_1)$ yields the regular network displaying

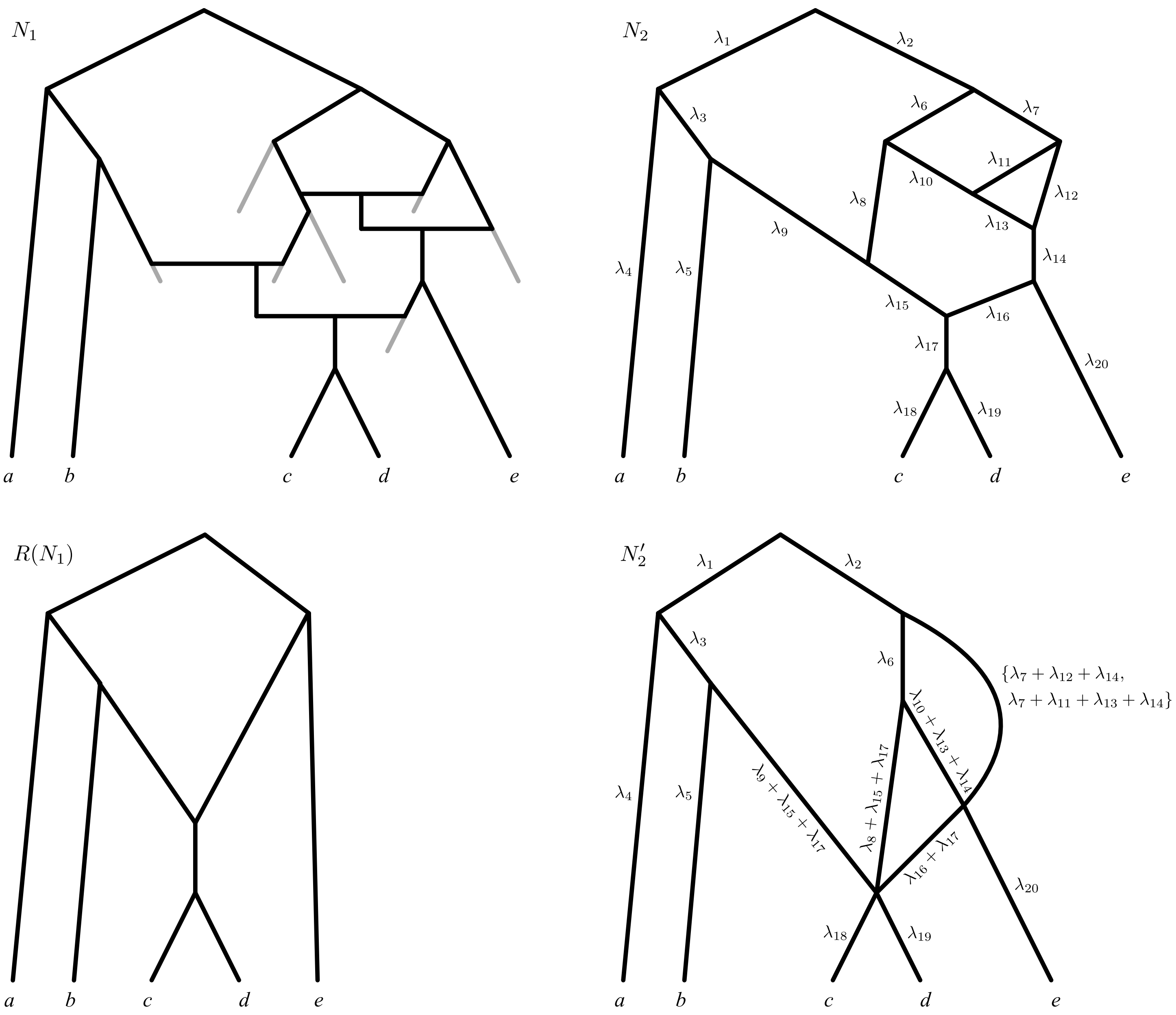

Fig 6. Comparison between the reduced version and the canonical form of a network. N_1 is the network topology in Fig. 15a of [46], where edges leading to extinct taxa are shown in grey, and reticulation events are represented by horizontal lines connecting the involved edges. N_2 is a phylogenetic network on the same set of taxa displaying the same evolutionary history, and showing edge lengths. $R(N_1)$ is the reduced version of N_1 (Fig. 15b of [46]). N'_2 is the canonical form of N_2. Comparing $R(N_1)$ and N'_2 reveals the difference in expressive power between reduced versions and canonical forms. Collapsing the edge above *c* and *d* in $R(N_1)$ yields the regular network displaying the same tree topologies as N_1 and N_2. Clearly, the reduced form $R(N_1)$ (and the regular form) retain less of the complexity of the original network N_1 than the canonical form N'_2. For example in $R(N_1)$ there remains no sign of the reticulate events ancestral to taxon *e*.

doi:10.1371/journal.pcbi.1004135.g006

the same tree topologies as N_1 and N_2. Again, this shows that the canonical form retains more of the complexity of the original network than its regular counterpart.

Results

Our main result consists of formally proving that for every network N there exists a network N' in canonical form, indistinguishable from N; moreover, if we restrict ourselves to networks

satisfying a mild condition (the NELP property below), such canonical form N' is unique (see Theorem 1). In other words, although in general a phylogenetic network N is not uniquely recoverable from the data it generates, there always exists a canonical version N' of N that is indeed determined by the data. Informally, N' is all that can be reconstructed about N.

In order to formally state this result, we here introduce a theoretical framework for explicit phylogenetic networks with branch lengths. A directed acyclic graph (*DAG*) is a simple directed graph that is free of directed cycles. A DAG is *rooted* if it contains precisely one node of indegree 0, called the *root*. All nodes of outdegree 0 in a DAG are called *leaves*. A *weighted rooted phylogenetic network* $N = (V, E, \varphi, \Lambda)$ on $\mathcal{X}$ (in this paper also called a *network* for simplicity) consists of a rooted DAG (V, E) whose leaves are bijectively labeled (via $\varphi{:}\mathcal{X} \rightarrow V$) with the elements of $\mathcal{X}$ (called *taxa*). Moreover, each edge $e \in E$ is associated to a set of positive weights, called *lengths*, $\Lambda(e) \subset \mathbb{R}_{>0}$. Figs. 3, 4, 5 contain examples of networks. A *reticulation* of a network N is a node $v \in V$ with indegree greater than 1. A *weighted phylogenetic tree* on $\mathcal{X}$ (a *tree* for simplicity) is a network on $\mathcal{X}$ with no reticulations and such that each edge e has a unique length ($|\Lambda(e)| = 1$), which we denote by $\lambda(e)$. Below, we discuss the biological justification of various aspects of the definitions above.

Let v be a node with indegree 1 and outdegree 1 in a tree. Node v is said to be *suppressible*. *Suppressing* v means removing the in-edge $e = (u, v)$ and the out-edge $f = (v, w)$ and then creating a new edge $g = (u, w)$ with length $\lambda(g) = \lambda(e) + \lambda(f)$. Let $N = (V, E, \varphi, \Lambda)$ be a network on $\mathcal{X}$. A *tree contained in* N is a tree $T = (V', E', \varphi', \lambda)$ on the same taxon set $\mathcal{X}$ such that: (1) the roots of T and N coincide, (2) the nodes and edges of T are also nodes and edges of N, that is $V' \subseteq V$ and $E' \subseteq E$, (3) taxon labels are unchanged, that is $\varphi' = \varphi$, and (4) the edge lengths of T are also edge lengths of N, that is, for every edge $e \in E'$, $\lambda(e) \in \Lambda(e)$. A *tree displayed by* N is a tree T' that can be obtained (up to isomorphism) by suppressing all suppressible nodes from a tree contained in N. The set of trees displayed by N is denoted by $\mathcal{T}(N)$. In Fig. 7, $\mathcal{T}(N_2')$ is the set of trees isomorphic to T_1' and T_2'. Two networks N_1 and N_2 are said to be *indistinguishable* if they

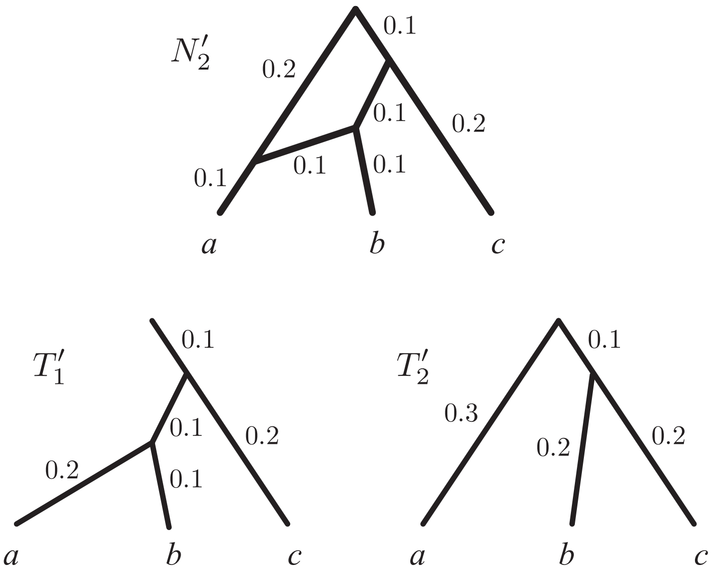

Fig 7. Trees displayed by a network. A rooted network N_2', and the trees it displays (T_1' and T_2'), obtained by removing a segment of length 0.5 from the outgroup lineage of N_2 in Fig. 2. In our formal setting, a network such as N_2 in Fig. 2 can either be represented as N_2' (by omitting the outgroup lineage, or part of it), or by rooting it in its outgroup (not shown).

doi:10.1371/journal.pcbi.1004135.g007

display the same set of trees, that is $\mathcal{T}(N_1) = \mathcal{T}(N_2)$. For example, N_1 and N_2 in Fig. 3 are indistinguishable, as they display the same set of trees (T_1, T_2 and T_3, up to isomorphism).

Definition 1. Given a network N, a *funnel* is a node with indegree greater than 0 and outdegree 1. A *funnel-free* network, or *canonical* network, is a network that does not contain funnels. A *canonical form* of a network N is a network that is funnel-free and indistinguishable from N.

In Fig. 3, N_1 and N_2 each contain two funnels, and thus are not funnel-free. The network N' in Fig. 4 is a canonical form of N_1 and N_2 in Fig. 3, as N' is funnel-free and indistinguishable from N_1 and N_2. Similarly, N_2' in Fig. 6 is a canonical form of N_2. Note that nodes with indegree 1 and outdegree 1 are funnels. This implies that for trees the funnel-free condition coincides with the exclusion of suppressible nodes, which is a standard requirement in the definition of phylogenetic trees. It is thus appropriate to view the funnel-free condition as a natural extension of this requisite to networks.

Definition 2. A *weighted path* in a network $N = (V, E, \varphi, \Lambda)$ is a pair (π, λ), where π is a directed path in the graph (V, E) and λ is a function that associates each edge e in π with a length $\lambda(e) \in \Lambda(e)$. The *length* of a weighted path is the sum of the lengths assigned to its edges. A network satisfies the *NELP* (*no equally long paths*) property if no pair of distinct weighted paths having the same endpoints have the same length.

As we explain below, the NELP property is a mild condition to satisfy, unless edge lengths are taken to represent time. The following result states that if we restrict ourselves to networks satisfying the NELP property, then every network has exactly one canonical form. An outline of its proof can be found in the Methods section, including an algorithm showing how to reduce a network to canonical form. The detailed proof is presented in S1 Text.

Theorem 1. *(i) Every network N has a canonical form. Moreover, (ii) if N has the NELP property, then there exists a unique canonical form of N among networks satisfying the NELP property (up to isomorphism).*

(The notion of isomorphism between networks is only used for mathematical rigor and is defined in S1 Text.) The following result provides a necessary and sufficient condition for two networks satisfying the NELP property to be indistinguishable.

Corollary 1. *Let N_1 and N_2 be networks with the NELP property and let N_1'* and *N_2' be their unique canonical forms satisfying the NELP property. Then N_1 and N_2 are indistinguishable if and only if N_1' and N_2' are the same network (up to isomorphism).*

The following result states that a canonical network with the NELP property is uniquely determined by the trees it displays:

Corollary 2. *Let N be a canonical network satisfying the NELP property. Then N is the unique (up to isomorphism) canonical network satisfying the NELP property that displays (all and only) the trees in $\mathcal{T}(N)$.*

We now discuss the biological significance of a number of technical aspects of our framework.

Definition of Networks and Trees Displayed by a Network

All the phylogenies considered here—trees or networks—are rooted. This is because we assume that the analysis uses an outgroup (possibly consisting of multiple taxa, and with no reticulations) for rooting. For simplicity, outgroup lineages are not included in our phylogenies (an exception to this is in Fig. 2). Note however that, because our phylogenies have edge lengths, and because omitting the outgroup is just a convention, the omitted lineages must have the same lengths for a network and all the trees it displays. For example, if we wish to omit the outgroup from N_2 in Fig. 2 and from the trees that it displays (T_1 and T_2 in Fig. 2), then what we obtain are N_2', T_1' and T_2' in Fig. 7. This has a notable consequence: the trees displayed by a rooted

network with edge lengths may have a root with outdegree 1 (e.g. T'_1 in Fig. 7). For flexibility, we also allow a network to have a root with outdegree 1.

Moreover, we allow multiple lengths for an edge in a network, but not in a tree. For example, in Fig. 6, network N'_2 has an edge with two lengths ($\lambda_7 + \lambda_{12} + \lambda_{14}$ and $\lambda_7 + \lambda_{11} + \lambda_{13} + \lambda_{14}$). The motivation behind multiple lengths lies in the observation that, whereas each edge in a phylogenetic tree describing the evolution of non-reticulating organisms trivially corresponds to a unique evolutionary path in the underlying real evolutionary history, when reticulate events have occurred this is not necessarily true: Fig. 8 and Fig. 9 show that some evolutionary scenarios can either be represented using multiedges (multiple edges with the same endpoints) or edges with multiple lengths. Although these two options are mathematically equivalent, graphically the second one leads to more compact representations, and this is why we choose to allow multiple lengths rather than multiedges. For our purposes we only need to consider the case where e has a finite set of lengths ($\Lambda(e) = \{\lambda_1(e), \ldots, \lambda_k(e)\}$).

Another unconventional aspect of our networks is the possibility of having nodes with in-degree and out-degree both greater than one. (See, e.g., the last common ancestor of c and d in N'_2 in Fig. 6.) Traditionally, the internal nodes in a phylogenetic network are constrained to belong to one of two different categories: reticulate nodes, with more than one incoming edge and just one outgoing edge, and speciation (or coalescence) nodes, with one incoming edge

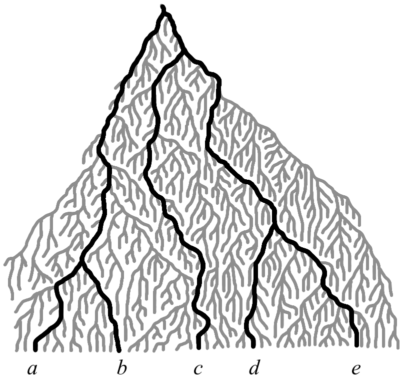

Fig 8. A non-reticulating evolutionary history (left) and a reticulating evolutionary history (right). The black lineages are those leading to a sampled set of taxa $\mathcal{X}$. The horizontal jagged lines represent reticulation events. Note that, whereas representing the scenario on the left with a phylogenetic tree on $\mathcal{X}$ is straightforward, for the one on the right several options are possible. We show three alternative representations in Fig. 9.

doi:10.1371/journal.pcbi.1004135.g008

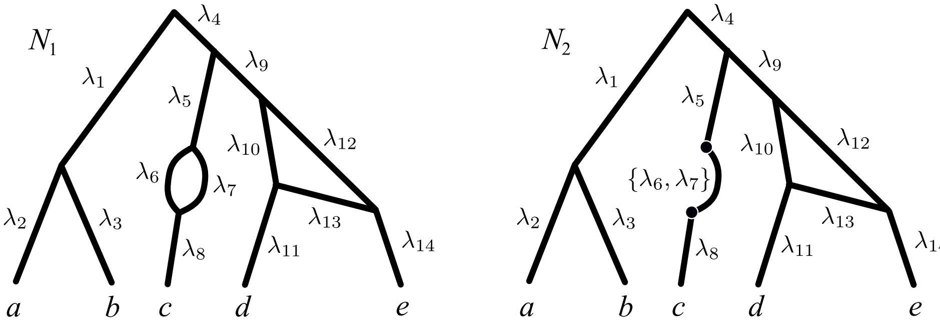

Fig 9. Alternative network representations for the evolutionary scenario in Fig. 8 (right). In our framework only N_2 is a network.

doi:10.1371/journal.pcbi.1004135.g009

and multiple outgoing edges. Because reticulate and speciation events are clearly distinct, it is reasonable to constrain internal nodes to only fall in the two categories above. In our framework, this requirement is dropped, and some networks, notably those in canonical form, may have nodes that both represent reticulate and speciation events. In this case, it is important to understand that these nodes represent a potentially complex (and unrecoverable) reticulate scenario, followed by one or more speciation events. Compare, for example, network N and its canonical form N' in Fig. 5, or N_2 and N_2' in Fig. 6. (In the latter, it is especially instructive to consider the reticulate history above the direct ancestor of taxon e.)

The NELP Property

We use network N_1 of Fig. 3 to illustrate the NELP property. In N_1 there are three distinct weighted paths having as endpoints the root of N_1 and the direct ancestor of b. The lengths of these paths are $\ell_1 = \lambda_1 + \lambda_6$, $\ell_2 = \lambda_2 + \lambda_3 + \lambda_5 + \lambda_8$ and $\ell_3 = \lambda_2 + \lambda_{10} + \lambda_9 + \lambda_8$. Moreover, there is another pair of paths having the same endpoints: those of lengths $\ell_4 = \lambda_3 + \lambda_5$ and $\ell_5 = \lambda_{10} + \lambda_9$. Thus N_1 has the NELP property if and only if the three numbers ℓ_1, ℓ_2 and ℓ_3 are all different (note that this implies that also ℓ_4 and ℓ_5 are different). If edge lengths are taken to represent evolutionary change, rather than time, this is a very mild requirement: when edge lengths are drawn at random from a continuous distribution, the probability that two paths get exactly the same length is zero.

On the other hand, the NELP property does not hold for phylogenetic networks where edge lengths are taken to represent time. For these networks, canonical forms may not be unique (see Fig. 10 for an example of this). Even in this case, we believe that inference methods should only consider phylogenetic networks in their canonical form, as this allows to reduce the solution space without any loss in "expressive power": since every network N has (at least one) canonical form that displays exactly the same set of trees—and therefore has the same fit with the data as N—restricting the solution space to canonical forms always leaves at least one optimal network within this space. The real weakness of using canonical forms in a molecular clock context is that if a canonical form is not unique, then it cannot be considered representative of all the networks indistinguishable from it. As an example of this, consider the indistinguishable networks in Fig. 10: none of these is representative of all the others.

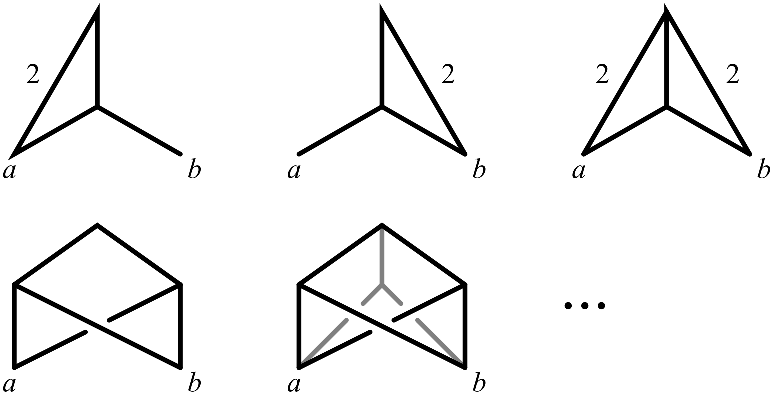

Fig 10. Different (non-isomorphic) but indistinguishable funnel-free networks. All edges are assumed to have the (unique) length 1 unless otherwise displayed. These networks do not satisfy the NELP property, showing that this is a necessary condition for the uniqueness of canonical forms (Theorem 1(ii)). The ellipsis at the end represents the fact that an infinite number of such networks can be obtained by adding any number of copies of the subgraph in grey in the last network.

doi:10.1371/journal.pcbi.1004135.g010

Discussion

Our results are both negative and positive. The bad news is that any method that scores the fit between a network *N* and the available data—which may be sequences, distances, splits, trees (with or without edge lengths)—based on the set of trees displayed by *N* must face an important theoretical limitation: regardless of the amount of available data from the taxa under consideration, some parts of the network representing their evolutionary history may be impossible to recover—most notably the relative order of consecutive reticulate events (see, e.g., Fig. 3). The good news is that, when edge lengths are taken into account, we can set precise limits to what is recoverable: the canonical form of a network *N* is a simplified version of *N* that excludes all the unrecoverable aspects of *N*. In a canonical form, reticulate events are brought as forward in time as possible, causing the collapse of multiple consecutive nodes. (Compare again network N_2 and its canonical form N_2' in Fig. 6.) The importance of the canonical form N' of a network *N* lies in the fact that, if we restrict our consideration to networks with the NELP property, N' is the unique canonical network consistent with perfect and unlimited data from the taxa in *N*.

There is an interesting analogy between soft polytomies in classical phylogenetics and collapsed nodes in a canonical network. Both represent lack of knowledge about the order of evolutionary events: speciations or more generally lineage splits in the first case, and reticulate events in the second. However, there is also an important difference between them: whereas in principle polytomies can be resolved by collecting further data from the taxa in the tree (for example, by extensive sequencing of their genomes [52]), the standard network inference methods considered here cannot resolve collapsed nodes in a canonical network, *irrespective of the amount of data from the taxa under consideration*. This difference is mitigated by the observation that increased taxon sampling may indeed permit to resolve the collapsed nodes, when the new lineages break adjacencies between reticulate nodes. However, such lineages may not always exist or they may be difficult to sample.

The present work has several consequences that should be of interest both to the biologists concerned by the use of methods for phylogenetic network inference, and to the researchers interested in the development of these methods. We illustrate these consequences starting from a well-known problem of network inference methods, that of multiple optima. It has been noted before that many of the inference methods that have been recently proposed—especially those solely based on topological features—often return multiple optimal networks: Huson and Scornavacca show a striking example of this (Fig. 2 in [53]), where the problem of finding the simplest network displaying two given tree topologies admits at least 486 optimal solutions.

The existence of multiple optimal networks for a given data set is essentially due to two reasons: *insufficient data* and *non-identifiability*. For the example of 486 optimal solutions, this large number may be partly due to the fact that the goal was to achieve consistency with only two tree topologies. More data may enable to discriminate among the 486 returned networks. Non-identifiability, which occurs when none of the allowed data can discriminate between two or more networks, is a more serious problem than insufficient data, as it cannot be solved by simply increasing the size of the input sample. Another interesting example appears in a paper by Albrecht et al. [54], which we reproduce here in Fig. 11. Here, there are only three optimal networks, essentially differing for which of the three clades {*A.bicornis, A.longissima, A.sharonensis*}, {*A.uniaristata, A.comosa*} and {*A.tauschii*} is considered as a hybrid (in this example reticulations represent hybridizations). This pattern is entirely analogous to that of the three networks in Fig. 2 (with *a*, *b* and *c* replaced by the three clades above), meaning that these three networks are indistinguishable to methods not accounting for edge lengths. Therefore, in this example, the existence of multiple optimal solutions is *entirely* due to non-identifiability.

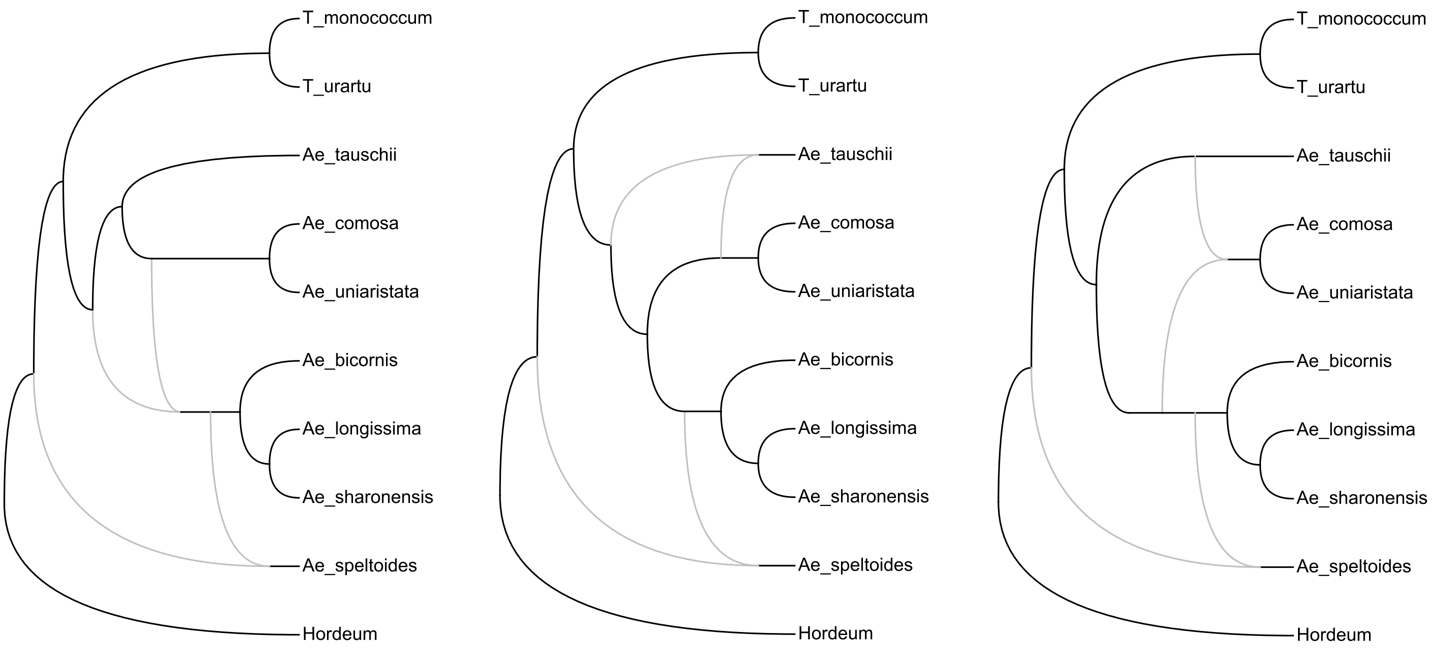

Fig 11. Real-world example of indistinguishable network topologies. (Reproduced from [54], Fig. 4.) Three network topologies that display the two tree topologies in Fig. 3 of [54]. Note that these three networks are analogous to N_1, N_2 and N_3 in Fig. 2 of the current paper: they each contain a reticulation cycle with three outgoing edges leading to the same three clades: {*A.bicornis, A.longissima, A.sharonensis*}, {*A.uniaristata, A.comosa*} and {*A.tauschii*} (in Fig. 2 instead of three clades we have three taxa *a*, *b* and *c*).

doi:10.1371/journal.pcbi.1004135.g011

All this motivates three recommendations:

1. It is important to use data in a way that causes non-identifiability to be as limited as possible. For example, as we have seen, accounting for edge lengths solves some cases of non-identifiability (e.g., in Fig. 2) although it does not eliminate this problem altogether (e.g., in Fig. 3).
2. Given an inferred network $\hat{N}$, it is important to know the set of networks that are theoretically impossible to distinguish from $\hat{N}$: no matter the amount of data, they will all receive the same support as $\hat{N}$. We may call this set the *indistinguishable class* of $\hat{N}$. The biologist using an inference method must be aware that $\hat{N}$ is not the only network supported by the data.
3. It would be highly useful to devise inference methods that instead of searching for (or directly constructing) solutions in the space of all possible networks, only considers one element per indistinguishable class. This has the potential to significantly speed up the inference.

Correspondingly, we recommend that edge lengths should be accounted for in the analyses (point 1) and, for each of the indistinguishable classes resulting from this choice, we identify a canonical network that, for all practical purposes, can be considered to be unique. Most important to the end users, we propose that a canonical network $\hat{N}$ is what should be given as the result of the inference, with the caveat that $\hat{N}$ is a way to represent a class of networks that are all equally supported (point 2). In a canonical form $\hat{N}$, the aspects that are not common to all networks in this class are collapsed, as described above. This will help the evolutionary biologist to

locate the uncertainties in the phylogeny, and possibly to choose further taxa to resolve them. Finally, we propose that inference methods only attempt to search among—or construct—phylogenetic networks in their canonical form (point 3).

We note that accounting for yet more characteristics of the data may reduce (or eliminate altogether) the identifiability issues for phylogenetic networks. In the case of sequence-based methods, one may take into account the natural order of sites within a sequence [11–13, 55, 56]. Similarly, for reconstruction methods based on collections of subtrees, one could observe and use the relative position of the different genomic regions supporting the input trees. However, these relative positions must be conserved across the genomes being analyzed, a condition which may hold for recombining organisms (e.g. individuals within a population or different viral strains), but which is not obvious when studying a group of taxa that have undergone reticulate events (e.g., hybridization) at some point in a distant past.

The main conclusion of the present study is the following: unless one abandons any optimization criterion that scores a network solely based on the trees it displays, the reconstruction should be carried out in a reduced space of networks: that of the canonical forms defined here. The motivation for this lies in the fact that canonical networks are guaranteed to be uniquely determined, if sufficient data are available. Once a canonical form $\hat{N}$ is inferred, it must be kept in mind that even assuming that the inference is free of statistical error, the true phylogenetic network is just one of the many networks having $\hat{N}$ as canonical form. Compared to what biologists are used to for phylogenetic trees—where in principle it is always possible to resolve uncertainties—it is clear that this requires an important change of perspective.

Methods

The following three subsections describe the proofs of Theorem 1 part (i), of Theorem 1 part (ii), and of their corollaries, respectively. In the case of Theorem 1 part (ii), only the gist of the proof is provided here. The proof in full detail is deferred to S1 Text.

Reduction Algorithm

In order to prove that any network N has a canonical form, we describe an algorithm to transform N into a canonical network indistinguishable from N. The algorithm simply consists of repeatedly applying to $N = (V, E, \varphi, \Lambda)$ one of the following two reduction rules, until neither can be executed (see Fig. 12):

Funnel suppression (R1). Given a funnel v with $k \geq 1$ in-edges $(u_1, v), (u_2, v), \ldots, (u_k, v)$ and out-edge (v, w), remove v and all these edges from N and introduce k new edges (u_1, w), $(u_2, w), \ldots, (u_k, w)$. For all $i \in \{1, 2, \ldots, k\}$ assign to (u_i, w) the lengths $\Lambda((u_i, w)) := \Lambda((u_i, v)) + \Lambda((v, w))$, where the sum of two sets of numbers A and B is defined as $A + B = \{a + b: a \in A, b \in B\}$.

Multiedge merging (R2). Given a collection of multi-edges (u, w) with multiplicity k and lengths $\Lambda'_1, \Lambda'_2, \ldots, \Lambda'_k$, replace these edges with a single edge with lengths $\bigcup_{i=1}^{k} \Lambda'_i$.

An example of the reduction of a network to its canonical form is shown in Fig. 13. Note that, even if the algorithm may temporarily produce multi-edges, the network produced in the end obviously does not have any multi-edge (otherwise we could still apply rule R2).

Proof of part (i) of Theorem 1. We must prove that any network $N = (V, E, \varphi, \Lambda)$ has a canonical form. For this, we apply the reduction algorithm described above, thus obtaining a sequence $N_0 = N, N_1, \ldots, N_m$, where each N_{i+1} is obtained from N_i by applying either R1 or R2. Neither R1 nor R2 can be applied to N_m. We prove that N_m is a canonical form of N. Although, strictly speaking, N_i may not be a network (as it potentially contains multi-edges), the notion of

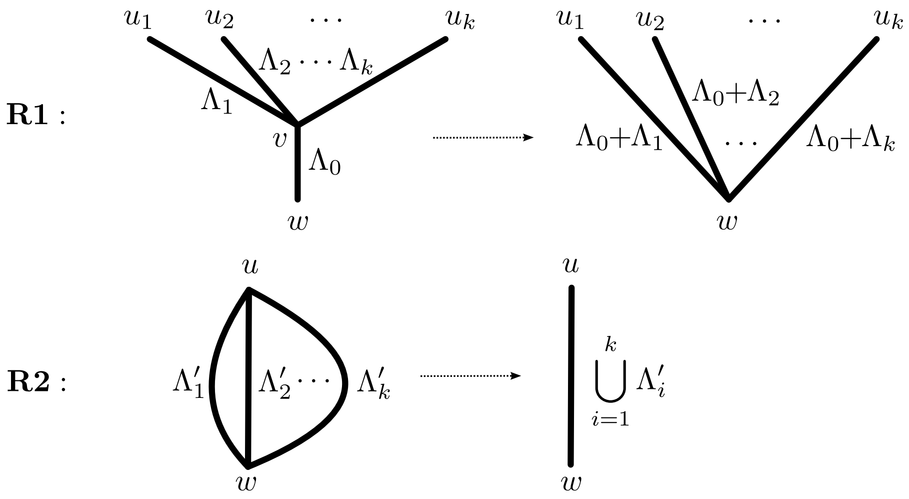

Fig 12. The two rules at the basis of the canonical reduction algorithm.

doi:10.1371/journal.pcbi.1004135.g012

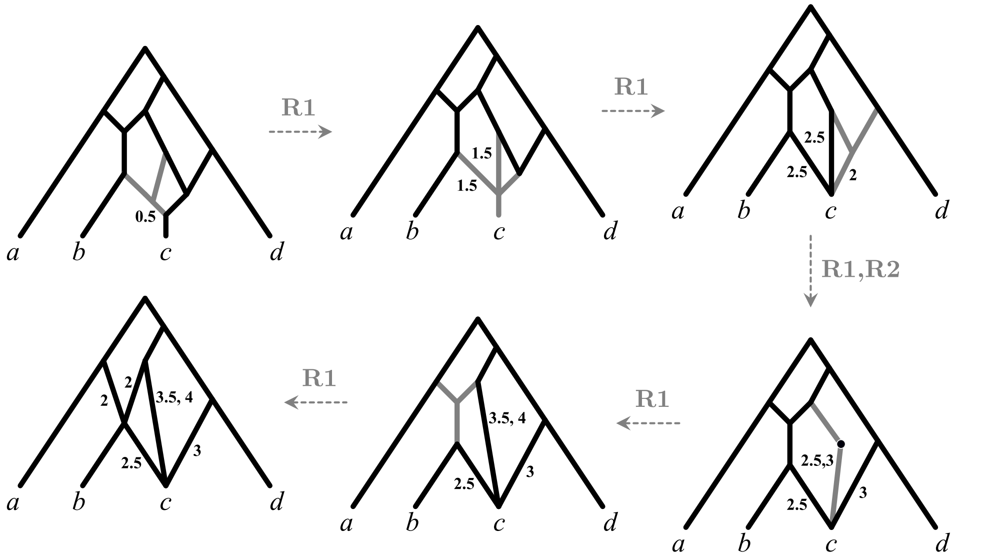

Fig 13. Reduction of a network to its canonical form. All edges are assumed to have the (unique) length 1 unless otherwise displayed. Gray edges are those to which the next reduction rule is applied.

doi:10.1371/journal.pcbi.1004135.g013

trees displayed by N_i, and thus that of indistinguishability, trivially extends to these multigraphs.

First, note that the algorithm terminates after a finite number of iterations (m). This is true because at each iteration the size of E is reduced by at least one. Moreover, the resulting network N_m is funnel-free, since no reduction of type R1 can be applied to it.

What is left to prove is that N_m is indistinguishable from $N = N_0$. To this end we prove that, at each iteration, N_i and N_{i+1} are indistinguishable, i.e. $\mathcal{T}(N_i) = \mathcal{T}(N_{i+1})$. In other words any tree T is displayed by N_i if and only if T is displayed by N_{i+1}.

Let T be displayed by N_i. Then T can be obtained by suppressing all suppressible nodes from a tree T_i contained in N_i. We consider three cases. (1) If none of the edges in T_i is involved

in the reduction transforming N_i into N_{i+1}, then clearly T_i is still contained in N_{i+1} and thus T is still displayed by N_{i+1}. (2) If T_i is involved in a R1 reduction, then it contains a funnel v and it contains one of the in-edges of the funnel, say (u_j, v), with length $\lambda_j \in \Lambda_j = \Lambda((u_j, v))$, along with the out-edge (v, w), with length $\lambda_0 \in \Lambda_0 = \Lambda((v, w))$. Now, let T_{i+1} be the tree obtained from T_i by suppressing the suppressible node v and thus creating a new edge (u_j, w) with length $\lambda_j + \lambda_0$. Because the R1 reduction creates a new edge (u_j, w) with length set $\Lambda_j + \Lambda_0$, containing the value $\lambda_j + \lambda_0$, then T_{i+1} is contained in N_{i+1}. Moreover, it easy to see that T can still be obtained by suppressing all suppressible nodes from T_{i+1}. Thus T is still displayed by N_{i+1}. (3) If T_i is involved in a R2 reduction, then it contains one of the edges of a multi-edge (u, w), with a length λ belonging to one of the length sets $\Lambda'_1, \Lambda'_2, \ldots, \Lambda'_k$ associated to the k copies of (u, w). Thus we have that $\lambda \in \bigcup_{i=1}^{k} \Lambda'_i$, which implies that T_i is still contained in N_{i+1} and thus T is still displayed by N_{i+1}. This concludes the proof of $\mathcal{T}(N_i) \subseteq \mathcal{T}(N_{i+1})$.

In order to prove that, conversely, $\mathcal{T}(N_{i+1}) \subseteq \mathcal{T}(N_i)$, one can proceed in a similar way as above: if T is displayed by N_{i+1}, then T can be obtained by suppressing all suppressible nodes from a tree T_{i+1} contained in N_i. By considering three cases analogous to the ones above regarding the involvement of T_{i+1} in the reduction transforming N_i into N_{i+1}, we can prove that in all these cases T is already displayed by N_i. Thus N_i and N_{i+1} are indistinguishable, which concludes our proof. □

We note informally that the order of application of the possible reductions in the algorithm above is irrelevant to the end result. To see this, it suffices to show that if two different reductions are applicable to a network, then the result of applying them is the same irrespective of the order of application. As we do not need this remark for the other results in this paper, we do not give a formal proof of it.

Lemma 1. Let N be a network and N′ a canonical form of N obtained by applying the reduction algorithm. If N satisfies the NELP property, then N′ satisfies the NELP property.

Proof: We prove that for each basic step of the reduction algorithm—transforming N_i into N_{i+1} via a reduction rule R1/R2—if N_i satisfies the NELP property, then N_{i+1} also satisfies it. Suppose the contrary; then, N_{i+1} contains two distinct weighted paths ρ_1, ρ_2 with the same endpoints u and v and same lengths. Because R1/R2 cannot create new nodes, u and v are also nodes in N_i. Moreover, it is easy to see that each weighted path ρ in N_i from u to v gives rise to exactly one weighted path $f(\rho)$ in N_{i+1} from u to v, with exactly the same length as ρ. Now take two weighted paths in N_i, one in the preimage $f^{-1}(\rho_1)$ and the other in the preimage $f^{-1}(\rho_2)$. These two weighted paths in N_i are distinct (as $\rho_1 \neq \rho_2$), have the same endpoints (u and v) and the same length. But then N_i violates the NELP property, leading to a contradiction. We thus have that if N_i satisfies the NELP property, then N_{i+1} also satisfies it. By iterating the argument above for each step in the reduction algorithm, the lemma follows. □

Uniqueness of the Canonical Form for Networks Satisfying the NELP

The proof of Theorem 1, part (ii), is rather technical. In this section, we introduce a number of new concepts and state the main intermediate results that are necessary to obtain this result. We leave their detailed proofs to S1 Text, together with the obvious definitions of basic concepts such as that of *isomorphic networks*, *sub-network* and *union* of two networks.

Definition 3. (*Root-leaf path, prefix, postfix, wishbone, crack.*) Let N be a network on $\mathcal{X}$ and (π, λ) be a weighted path in N from the root of N to a leaf labelled by $x \in \mathcal{X}$. Now consider the sub-network $P = (V(\pi), E(\pi), \varphi|_{\{x\}}, \lambda)$ on $\{x\}$ consisting of all the nodes and edges in π and associated labels. Any sub-network of N such as P is called a *root-leaf path* of N. Given a root-leaf path P and a node v belonging to it, any weighted path formed by all the ancestors [descendants] of v in P is a *prefix* [*suffix*] of P. Note that a prefix [suffix] only consists of one node

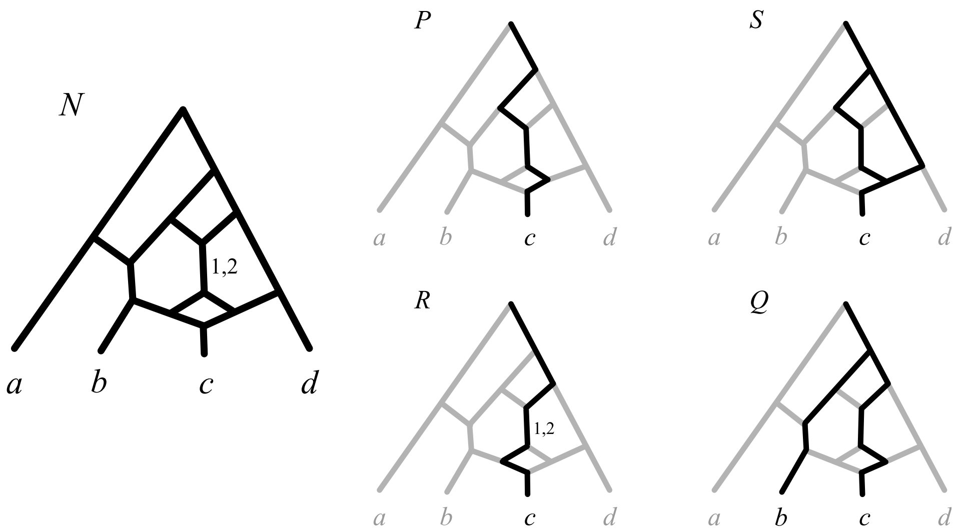

Fig 14. Illustration of Definition 3. *P* (edges in black) is a root-leaf path of *N* and thus both a wishbone and a crack of *N*. *R* and *S* (black) are cracks of *N*. *Q* (black) is a wishbone of *N*. All edges are assumed to have the (unique) length 1 unless otherwise displayed.

doi:10.1371/journal.pcbi.1004135.g014

when *v* is the root [leaf] of *P*. A *wishbone* of *N* is any sub-network of *N* formed by taking the union of two root-leaf paths that have in common only a prefix. A *crack* of *N* is any sub-network of *N* formed by taking the union of two root-leaf paths that have in common only a prefix and a suffix.

Fig. 14 illustrates the definitions above. Note that any root-leaf path *P* is both a wishbone and a crack, as *P* is the result of the union of *P* with itself, and *P* has a common prefix and a common suffix with *P*. Moreover, any sub-network *R* that can be obtained from a root-leaf path by attributing two lengths to one of its edges *e* is a crack. Finally, note that wishbones and cracks are networks, and thus the notion of isomorphism (Definition 5 in S1 Text) can be applied to them.

The proof of part (ii) in Theorem 1 depends on two important results (Propositions 1 and 2 below), whose proofs can be found in S1 Text. The first states that a network with the NELP property is uniquely determined by the wishbones and cracks it contains.

Proposition 1. Two networks N_1 and N_2 with the NELP property are isomorphic if and only if they contain the same wishbones and cracks (up to isomorphism).

Proposition 1 is interesting on its own as it suggests an enumerative algorithm to verify whether two networks with the NELP property are isomorphic. Unfortunately this algorithm would be impractical, as the number of wishbones (or cracks) in a network is not polynomial in the size of the network. Also note that we require N_1 and N_2 to satisfy the NELP property because there exist non-isomorphic networks containing the same wishbones and cracks: for example the networks in the bottom line of Fig. 10. The second result that we need is the following:

Proposition 2. Let N_1 and N_2 be two indistinguishable funnel-free networks, satisfying the NELP property. Then they contain the same wishbones and cracks (up to isomorphism).

Proof of part (ii) of Theorem 1. Let *N* be a network with the NELP property and N' a canonical form of *N* obtained by applying the reduction algorithm. By Lemma 1, N' satisfies the NELP property. Now suppose that there exists another canonical form of *N*, called N'', satisfying the NELP property. By transitivity, N' and N'' are indistinguishable. Because N' and N'' are indistinguishable, funnel-free and with the NELP property, N' and N'' must contain the same

wishbones and cracks (because of Proposition 2). But then, because of Proposition 1, N' and N'' are isomorphic. □

We note that some of our arguments in S1 Text lead us to conjecture that a funnel-free network satisfying the NELP property cannot be indistinguishable from a funnel-free network violating the NELP property. This claim would allow us to simplify the statement of Theorem 1: networks with the NELP property would be guaranteed to have a unique canonical form (not just among networks with the NELP property, but among *all* networks). Unfortunately, to this date, we were unable to prove this conjecture. Nonetheless, note that the reduction algorithm returns, for any network with the NELP property, its *unique* canonical form with the NELP property (by Lemma 1).

Corollaries

It remains to prove the two corollaries at the end of the Results section. The first one states that two networks N_1 and N_2 satisfying the NELP property are indistinguishable if and only if their unique canonical forms with the NELP property, N_1' and N_2' respectively, are isomorphic. By Lemma 1, N_1' and N_2' can be obtained by applying the reduction algorithm to N_1 and N_2.

Proof of Corollary 1. The *if* part trivially follows from the transitivity of indistinguishability. As for the *only if* part, note that (again by transitivity) N_1' is indistinguishable from N_2. As it is also funnel-free, N_1' is a canonical form of N_2. Because N_2 can only have one canonical form satisfying the NELP property (by Theorem 1 (ii)), N_1' and N_2' must be the same network (up to isomorphism). □

As for Corollary 2, we recall that it states that a canonical network N with the NELP property is uniquely determined by the trees it displays.

Proof of Corollary 2. Let N and N' be indistinguishable canonical networks satisfying the NELP property. Then, N and N' are both canonical forms of N satisfying the NELP. But then, by Theorem 1(ii), N and N' must be the same network (up to isomorphism). □

Supporting Information

S1 Text. Supporting Information: a mathematical theory of explicit phylogenetic networks with edge lengths. This document provides an introduction to the mathematical theory of explicit phylogenetic networks with edge lengths, leading in particular to the proofs of Propositions 1 and 2, which are necessary for the proof of Theorem 1, part (ii). In the last section, we consider networks with inheritance probabilities and their relevance for likelihood-based reconstruction.
(PDF)

Acknowledgments

We are grateful to O.Gascuel for advice on the structure of the paper.

Author Contributions

Wrote the paper: FP CS. Conceived the question: FP CS. Proved the main formal results: FP.

References

1. Huson DH, Rupp R, Scornavacca C (2011) Phylogenetic Networks: Concepts, Algorithms and Applications. Cambridge University Press.
2. Nakhleh L (2011) Evolutionary phylogenetic networks: models and issues. In: The Problem Solving Handbook in Computational Biology and Bioinformatics, Springer. pp. 125–158.

3. Morrison DA (2011) Introduction to Phylogenetic Networks. RJR Productions.
4. Mallet J (2007) Hybrid speciation. Nature 446: 279–283. doi: 10.1038/nature05706 PMID: 17361174
5. Nolte AW, Tautz D (2010) Understanding the onset of hybrid speciation. Trends in Genetics 26: 54–58. doi: 10.1016/j.tig.2009.12.001 PMID: 20044166
6. Ochman H, Lawrence J, Groisman E (2000) Lateral gene transfer and the nature of bacterial innovation. Nature 405: 299–304. doi: 10.1038/35012500 PMID: 10830951
7. Boto L (2010) Horizontal gene transfer in evolution: facts and challenges. Proceedings of the Royal Society B: Biological Sciences 277: 819–827. doi: 10.1098/rspb.2009.1679 PMID: 19864285
8. Smith GJ, Vijaykrishna D, Bahl J, Lycett SJ, Worobey M, et al. (2009) Origins and evolutionary genomics of the 2009 swine-origin H1N1 influenza A epidemic. Nature 459: 1122–1125. doi: 10.1038/nature08182 PMID: 19516283
9. Rambaut A, Posada D, Crandall K, Holmes E (2004) The causes and consequences of HIV evolution. Nature Reviews Genetics 5: 52–61. doi: 10.1038/nrg1246 PMID: 14708016
10. Simon-Loriere E, Holmes EC (2011) Why do RNA viruses recombine? Nature Reviews Microbiology 9: 617–626. doi: 10.1038/nrmicro2614 PMID: 21725337
11. Song YS, Hein J (2005) Constructing minimal ancestral recombination graphs. Journal of Computational Biology 12: 147–169. doi: 10.1089/cmb.2005.12.147 PMID: 15767774
12. Minichiello M, Durbin R (2006) Mapping trait loci by use of inferred ancestral recombination graphs. American Journal of Human Genetics 79: 910–922. doi: 10.1086/508901 PMID: 17033967
13. Rasmussen MD, Hubisz MJ, Gronau I, Siepel A (2014) Genome-wide inference of ancestral recombination graphs. PLoS Genetics 10: e1004342. doi: 10.1371/journal.pgen.1004342 PMID: 24831947
14. Huson D, Bryant D (2006) Application of phylogenetic networks in evolutionary studies. Molecular Biology and Evolution 23: 254–267. doi: 10.1093/molbev/msj030 PMID: 16221896
15. Bryant D, Moulton V (2004) Neighbor-net: An agglomerative method for the construction of phylogenetic networks. Molecular Biology and Evolution 21: 255–265. doi: 10.1093/molbev/msh018 PMID: 14660700
16. Huson DH, Richter DC, Rausch C, Dezulian T, Franz M, et al. (2007) Dendroscope: An interactive viewer for large phylogenetic trees. BMC Bioinformatics 8: 460. doi: 10.1186/1471-2105-8-460 PMID: 18034891
17. Hallström BM, Kullberg M, Nilsson MA, Janke A (2007) Phylogenomic data analyses provide evidence that Xenarthra and Afrotheria are sister groups. Molecular Biology and Evolution 24: 2059–2068. doi: 10.1093/molbev/msm136 PMID: 17630282
18. Lorentz Center (2012). The future of phylogenetic networks. Available: http://www.lorentzcenter.nl/lc/web/2012/515/description.php3?wsid = 515. Accessed 20 Oct 2014.
19. Bapteste E, van Iersel L, Janke A, Kelchner S, Kelk S, et al. (2013) Networks: expanding evolutionary thinking. Trends in Genetics 29: 439–441. doi: 10.1016/j.tig.2013.05.007 PMID: 23764187
20. Morrison D (2013). What are evolutionary networks currently used for? Available: http://phylonetworks.blogspot.fr/2013/10/what-are-evolutionary-networks.html. Accessed 20 Oct 2014.
21. Delwiche CF, Palmer JD (1996) Rampant horizontal transfer and duplication of rubisco genes in eubacteria and plastids. Molecular Biology and Evolution 13: 873–882. doi: 10.1093/oxfordjournals.molbev.a025647 PMID: 8754222
22. Morgan DR (2003) nrDNA external transcribed spacer (ETS) sequence data, reticulate evolution, and the systematics of Machaeranthera (Asteraceae). Systematic Botany 28: 179–190.
23. Marhold K, Lihová J (2006) Polyploidy, hybridization and reticulate evolution: lessons from the Brassicaceae. Plant Systematics and Evolution 259: 143–174. doi: 10.1007/s00606-006-0417-x
24. Koblmüller S, Duftner N, Sefc KM, Aibara M, Stipacek M, et al. (2007) Reticulate phylogeny of gastropod-shell-breeding cichlids from Lake Tanganyika—the result of repeated introgressive hybridization. BMC Evolutionary Biology 7: 7. doi: 10.1186/1471-2148-7-7 PMID: 17254340
25. Richards TA, Soanes DM, Foster PG, Leonard G, Thornton CR, et al. (2009) Phylogenomic analysis demonstrates a pattern of rare and ancient horizontal gene transfer between plants and fungi. The Plant Cell 21: 1897–1911. doi: 10.1105/tpc.109.065805 PMID: 19584142
26. Dyer RJ, Savolainen V, Schneider H (2012) Apomixis and reticulate evolution in the Asplenium monanthes fern complex. Annals of Botany 110: 1515–1529. doi: 10.1093/aob/mcs202 PMID: 22984165
27. Thiergart T, Landan G, Schenk M, Dagan T, Martin WF (2012) An evolutionary network of genes present in the eukaryote common ancestor polls genomes on eukaryotic and mitochondrial origin. Genome Biology and Evolution 4: 466–485. doi: 10.1093/gbe/evs018 PMID: 22355196

28. Huson D, Scornavacca C (2011) A survey of combinatorial methods for phylogenetic networks. Genome Biology and Evolution 3: 23. doi: 10.1093/gbe/evq077 PMID: 21081312
29. Jin G, Nakhleh L, Snir S, Tuller T (2006) Efficient parsimony-based methods for phylogenetic network reconstruction. In: Proceedings of the 5th European Conference on Computational Biology (ECCB). volume 23 of *Bioinformatics*, pp. e123–e128.
30. Jin G, Nakhleh L, Snir S, Tuller T (2007) Inferring phylogenetic networks by the maximum parsimony criterion: A case study. Molecular Biology and Evolution 24: 324–337. doi: 10.1093/molbev/msl163 PMID: 17068107
31. Jin G, Nakhleh L, Snir S, Tuller T (2009) Parsimony score of phylogenetic networks: hardness results and a linear-time heuristic. IEEE/ACM Transactions on Computational Biology and Bioinformatics 6: 495–505. doi: 10.1109/TCBB.2008.119 PMID: 19644176
32. Jin G, Nakhleh L, Snir S, Tuller T (2006) Maximum likelihood of phylogenetic networks. Bioinformatics 22: 2604–2611. doi: 10.1093/bioinformatics/btl452 PMID: 16928736
33. Park HJ, Nakhleh L (2012) Inference of reticulate evolutionary histories by maximum likelihood: the performance of information criteria. BMC Bioinformatics 13: S12.
34. van Iersel L, Kelk S, Rupp R, Huson D (2010) Phylogenetic networks do not need to be complex: Using fewer reticulations to represent conflicting clusters. In: Proceedings of the 18th Annual International Conference on Intelligent Systems for Molecular Biology (ISMB). volume 26 of *Bioinformatics*, pp. i124–i131.
35. To TH, Habib M (2009) Level-k phylogenetic networks are constructable from a dense triplet set in polynomial time. In: Combinatorial Pattern Matching: Proceeding of the 20th Annual Symposium Combinatorial Pattern Matching (CPM). volume 5577 of *LNCS*, pp. 275–288.
36. Grünewald S, Forslund K, Dress A, Moulton V (2007) Qnet: An agglomerative method for the construction of phylogenetic networks from weighted quartets. Molecular Biology and Evolution 24: 532–538. PMID: 17119010
37. Baroni M, Semple C, Steel M (2006) Hybrids in real time. Systematic Biology 55: 46–56. doi: 10.1080/10635150500431197 PMID: 16507523
38. Yu Y, Degnan JH, Nakhleh L (2012) The probability of a gene tree topology within a phylogenetic network with applications to hybridization detection. PLoS genetics 8: e1002660. doi: 10.1371/journal.pgen.1002660 PMID: 22536161
39. Radice R (2011) A Bayesian Approach to Phylogenetic Networks. Ph.D. thesis, University of Bath.
40. Gusfield D, Eddhu S, Langley C (2003) Efficient reconstruction of phylogenetic networks with constrained recombinations. In: Proceedings of the IEEE Computer Society Conference on Bioinformatics (CSB). IEEE Computer Society, p. 363.
41. Wang L, Zhang K, Zhang L (2001) Perfect phylogenetic networks with recombination. Journal of Computational Biology 8: 69–78. doi: 10.1089/106652701300099119 PMID: 11339907
42. Huson DH, Rupp R, Berry V, Gambette P, Paul C (2009) Computing galled networks from real data. Bioinformatics 25: i85–i93. doi: 10.1093/bioinformatics/btp217 PMID: 19478021
43. Choy C, Jansson J, Sadakane K, Sung WK (2005) Computing the maximum agreement of phylogenetic networks. Theoretical Computer Science 335: 93–107. doi: 10.1016/j.tcs.2004.12.012
44. Cardona G, Rosselló F, Valiente G (2007) Comparison of tree-child phylogenetic networks. IEEE/ACM Transactions on Computational Biology and Bioinformatics 6: 552–569. doi: 10.1109/TCBB.2007.70270
45. Cardona G, Llabrés M, Rosselló F, Valiente G (2008) A distance metric for a class of tree-sibling phylogenetic networks. Bioinformatics 24: 1481–1488. doi: 10.1093/bioinformatics/btn231 PMID: 18477576
46. Moret B, Nakhleh L, Warnow T, Linder C, Tholse A, et al. (2004) Phylogenetic networks: modeling, reconstructibility, and accuracy. IEEE Transactions on Computational Biology and Bioinformatics 1: 13–23. doi: 10.1109/TCBB.2004.10 PMID: 17048405
47. Nakhleh L (2010) A metric on the space of reduced phylogenetic networks. IEEE/ACM Transactions on Computational Biology and Bioinformatics 7: 218–222. doi: 10.1109/TCBB.2009.2 PMID: 20431142
48. Baroni M, Semple C, Steel MA (2004) A framework for representing reticulate evolution. Annals of Combinatorics 8: 391–408. doi: 10.1007/s00026-004-0228-0
49. Gambette P, Huber KT (2012) On encodings of phylogenetic networks of bounded level. Journal of Mathematical Biology 65: 157–180. doi: 10.1007/s00285-011-0456-y PMID: 21755321
50. Cardona G, Rosselló F, Valiente G (2008) Tripartitions do not always discriminate phylogenetic networks. Mathematical Biosciences 211: 356–370. doi: 10.1016/j.mbs.2007.11.003 PMID: 18177903

51. Willson SJ (2011) Regular networks can be uniquely constructed from their trees. IEEE/ACM Transactions on Computational Biology and Bioinformatics 8: 785–796. doi: 10.1109/TCBB.2010.69 PMID: 20714025

52. Delsuc F, Brinkmann H, Philippe H (2005) Phylogenomics and the reconstruction of the tree of life. Nature Reviews Genetics 6: 361–375. doi: 10.1038/nrg1603 PMID: 15861208

53. Huson DH, Scornavacca C (2012) Dendroscope 3: An interactive tool for rooted phylogenetic trees and networks. Systematic Biology 61: 1061–1067. doi: 10.1093/sysbio/sys062 PMID: 22780991

54. Albrecht B, Scornavacca C, Cenci A, Huson DH (2012) Fast computation of minimum hybridization networks. Bioinformatics 28: 191–197. doi: 10.1093/bioinformatics/btr618 PMID: 22072387

55. Hein J (1993) A heuristic method to reconstruct the history of sequences subject to recombination. Journal of Molecular Evolution 36: 396–405. doi: 10.1007/BF00182187

56. Snir S, Tuller T (2009) The Net-HMM approach: Phylogenetic network inference by combining maximum likelihood and hidden Markov models. Journal of Bioinformatics and Computational Biology 7: 625–644. doi: 10.1142/S021972000900428X PMID: 19634195
